# Cyclic Vomiting Syndrome in Children

**DOI:** 10.3389/fneur.2020.583425

**Published:** 2020-11-02

**Authors:** Umberto Raucci, Osvaldo Borrelli, Giovanni Di Nardo, Renato Tambucci, Piero Pavone, Silvia Salvatore, Maria Elisabetta Baldassarre, Duccio Maria Cordelli, Raffaele Falsaperla, Enrico Felici, Michela Ada Noris Ferilli, Salvatore Grosso, Saverio Mallardo, Diego Martinelli, Paolo Quitadamo, Licia Pensabene, Claudio Romano, Salvatore Savasta, Alberto Spalice, Caterina Strisciuglio, Agnese Suppiej, Massimiliano Valeriani, Letizia Zenzeri, Alberto Verrotti, Annamaria Staiano, Maria Pia Villa, Martino Ruggieri, Pasquale Striano, Pasquale Parisi

**Affiliations:** ^1^Pediatric Emergency Department, Bambino Gesù Children's Hospital, Institute for Research, Hospitalization and Health Care (IRCCS), Rome, Italy; ^2^Division of Neurogastroenterology and Motility, Department of Pediatric Gastroenterology, University College London (UCL) Institute of Child Health and Great Ormond Street Hospital, London, United Kingdom; ^3^Chair of Pediatrics, Department of Neuroscience, Mental Health and Sense Organs (NESMOS), Faculty of Medicine & Psychology, Sant'Andrea Hospital, Sapienza University of Rome, Rome, Italy; ^4^Digestive Endoscopy and Surgery Unit, Bambino Gesù Children's Hospital, Institute for Research, Hospitalization and Health Care (IRCCS), Rome, Italy; ^5^Section of Pediatrics and Child Neuropsychiatry, Department of Clinical and Experimental Medicine, University of Catania, Catania, Italy; ^6^Pediatric Department, Ospedale “F. Del Ponte,” University of Insubria, Varese, Italy; ^7^Department of Biomedical Science and Human Oncology Aldo Moro University of Bari, Bari, Italy; ^8^Child Neurology Unit, University of Bologna, Bologna, Italy; ^9^Neonatal Intensive Care and Pediatric Units, S. Marco Hospital, Vittorio Emanuele Hospital, Catania, Italy; ^10^Unit of Pediatrics, The Children Hospital, Azienda Ospedaliera SS Antonio e Biagio e Cesare Arrigo, Alessandria, Italy; ^11^Division of Neurology, Bambino Gesù Children's Hospital, Institute for Research, Hospitalization and Health Care (IRCCS), Rome, Italy; ^12^Clinical Pediatrics, Department of Molecular Medicine and Development, University of Siena, Siena, Italy; ^13^Pediatric Department, Santa Maria Goretti Hospital, Sapienza University of Rome, Latina, Italy; ^14^Division of Metabolism, Department of Pediatric Specialties, Bambino Gesù Children's Hospital, Institute for Research, Hospitalization and Health Care (IRCCS), Rome, Italy; ^15^Department of Pediatrics, A.O.R.N. Santobono-Pausilipon, Naples, Italy; ^16^Pediatric Unit, Department of Medical and Surgical Sciences, University “Magna Graecia” of Catanzaro, Catanzaro, Italy; ^17^Pediatric Gastroenterology Unit, Department of Human Pathology in Adulthood and Childhood “G. Barresi”, University of Messina, Messina, Italy; ^18^Pediatric Unit “Hospital ASST of Crema,” Crema, Italy; ^19^Child Neurology Division, Department of Pediatrics, “Sapienza,” University of Rome, Rome, Italy; ^20^Department of Woman, Child, General and Specialistic Surgery, University of Campania “Luigi Vanvitelli,” Naples, Italy; ^21^Pediatric Section, Department of Medical Sciences, University of Ferrara, Ferrara, Italy; ^22^Emergency Pediatric Department, Santobono-Pausilipon Children's Hospital, Naples, Italy; ^23^Department of Pediatrics, University of L'Aquila, L'Aquila, Italy; ^24^Section of Pediatrics, Department of Translational Medical Science, “Federico II” University of Naples, Naples, Italy; ^25^Unit of Rare Diseases of the Nervous System in Childhood, Section of Pediatrics and Child Neuropsychiatry, Department of Clinical and Experimental Medicine, University of Catania, Catania, Italy; ^26^Department of Neurosciences, Rehabilitation, Ophthalmology, Genetics, Maternal and Child Health, University of Genoa, Genova, Italy; ^27^Institute for Research, Hospitalization and Health Care (IRCCS) “G. Gaslini” Institute, Genova, Italy

**Keywords:** functional gastrointestinal disorders, migraine, vomiting, antiemetics, anticonvulsants, cyclic vomiting syndrome, differential diagnosis, episodic syndromes that may be associated with migraine

## Abstract

Cyclic Vomiting Syndrome (CVS) is an underdiagnosed episodic syndrome characterized by frequent hospitalizations, multiple comorbidities, and poor quality of life. It is often misdiagnosed due to the unappreciated pattern of recurrence and lack of confirmatory testing. CVS mainly occurs in pre-school or early school-age, but infants and elderly onset have been also described. The etiopathogenesis is largely unknown, but it is likely to be multifactorial. Recent evidence suggests that aberrant brain-gut pathways, mitochondrial enzymopathies, gastrointestinal motility disorders, calcium channel abnormalities, and hyperactivity of the hypothalamic-pituitary-adrenal axis in response to a triggering environmental stimulus are involved. CVS is characterized by acute, stereotyped and recurrent episodes of intense nausea and incoercible vomiting with predictable periodicity and return to baseline health between episodes. A distinction with other differential diagnoses is a challenge for clinicians. Although extensive and invasive investigations should be avoided, baseline testing toward identifying organic causes is recommended in all children with CVS. The management of CVS requires an individually tailored therapy. Management of acute phase is mainly based on supportive and symptomatic care. Early intervention with abortive agents during the brief prodromal phase can be used to attempt to terminate the attack. During the interictal period, non-pharmacologic measures as lifestyle changes and the use of reassurance and anticipatory guidance seem to be effective as a preventive treatment. The indication for prophylactic pharmacotherapy depends on attack intensity and severity, the impairment of the QoL and if attack treatments are ineffective or cause side effects. When children remain refractory to acute or prophylactic treatment, or the episode differs from previous ones, the clinician should consider the possibility of an underlying disease and further mono- or combination therapy and psychotherapy can be guided by accompanying comorbidities and specific sub-phenotype. This review was developed by a joint task force of the Italian Society of Pediatric Gastroenterology Hepatology and Nutrition (SIGENP) and Italian Society of Pediatric Neurology (SINP) to identify relevant current issues and to propose future research directions on pediatric CVS.

## Introduction

Cyclic Vomiting Syndrome (CVS) is identified by acute, stereotyped and recurrent episodes of intense nausea with incoercible vomiting, lasting from a few hours to a few days; both children and adults are affected, although the clinical presentation and natural history vary somewhat with age (1). CVS was first described in 1806 by Heberden (2) and then by Gee in the St. Bartholomew's Hospital Reports (3). Since pediatric CVS evolves into migraine later in life in most patients and based on a high family prevalence of migraines, the effectiveness of anti-migraine therapy and observation of mitochondrial DNA polymorphisms in CVS and migraine patients, CVS has been considered a migraine-related or migraine-equivalent disorder (1, 4, 5). In the International Classification of Headache Disorders (ICHD III beta) (6) considers SVC as a pediatric migraine variant among the episodic syndromes that may be associated with migraine. The recent Rome IV Criteria included CVS among the “functional gastrointestinal disorders” (FGID), idiopathic disorders of gut-brain interaction affecting different parts of the gastrointestinal tract symptoms that are not attributable to organic etiology (7–9).

The etiopathogenesis is likely to be multifactorial. Recent evidence suggests that aberrant brain-gut pathways, mitochondrial enzymopathies, gastrointestinal motility disorders, calcium channel abnormalities, and hyperactivity of the hypothalamic-pituitary-adrenal axis in response to a triggering environmental stimulus are involved in the CVS development (10). Genetic factors have been linked to CVS, but further research is required to better establish the heritable basis of this disorder (11).

This review was developed by a joint task force of the Italian Society of Pediatric Gastroenterology Hepatology and Nutrition (SIGENP) and Italian Society of Pediatric Neurology (SINP) to propose future research directions.

## Epidemiology

There are difficulties in obtaining reliable epidemiological evidence for CVS, being an undiagnosed condition (12). In children, a prevalence of 1.9% has been reported by two school-based surveys from Scotland and Turkey (13, 14), while the incidence of new pediatric cases was 3.15 per 100,000 children per year in an Irish population-based study (15). In a primary care cross-sectional, among Colombian children aged 0–48 months, ~0.5–7% of them received CVS diagnosis (16). Although mainly occurs in pre-school or early school-age, CVS appears to be more common in adults than previously thought (12, 17–19), and delayed diagnosis has been reported. Indeed, patients are frequently misdiagnosed as having recurrent gastroenteritis, food poisoning, and eating disorders (20). In a study from the U.S. mean ages at onset of symptoms and diagnosis were 5.7 ± 0.3 and 8.0 ± 0.3 years (21).

Patients with CVS are predominantly white, followed by African American and Hispanic (22, 23). A recent nationwide analysis conducted in US of over 20,000 adults hospitalized for CVS showed that 63% of patients were white, 18% were African American, and 6% were Hispanic (24). Moreover, CVS appears to be slightly more common in female (13–15, 18, 21, 25, 26) and is associated with family (especially maternal) or personal history of migraines (up to 82%) (27, 28). The highly documented later development of migraine (up to 75% of children) suggests a progressive continuum from CVS to migraine headaches in most children (13, 27, 29–31). Nearly 60% of children outgrow CVS (29) with a reported median overall duration of the disorder of 66 months (range 3–179) (30). CVS determines a worsening in quality of life of children, needing multiple hospitalizations for acute dehydration, missing a mean of 20 days of school each year (32) with an annual cost of ~$ 17,035 per individual patient (1).

## Pathophysiology

The pathophysiology of CVS is yet to be established although several potential underlying mechanisms have been postulated. The emetic reflex is highly complex, and its final common pathway and its central mechanisms have yet to be fully elucidated. It is widely accepted that several nuclei within the medulla oblongata between the obex and the rostral portion of the nucleus ambiguous play a key role in the central coordination of emetic neurocircuitry (33). Among these nuclei, which collectively are conceptualized as a central pattern generator, the nucleus tractus solitarius (NTS) within the dorsal vagal complex (DVC) represents the main integrative site for modulation of the emetic reflex. Activation of NTS to evoke vomiting occurs via inputs from the GI tract and other visceral organs via the vagus nerve, vestibular system, and higher brain regions including the cerebral cortex, hypothalamus, cerebellum, and the *area postrema* (AP). The latter, defined as chemoreceptor trigger zone (CTZ), is an important component of emetic arc and is located in the floor of the fourth ventricle outside the blood-brain barrier with the potential to detect circulating toxin. Distinct neural input from NTS coordinates the motor pathways driving the visceral and somatic motor events of vomiting by activating nuclei within the hindbrain in a precisely synchronized temporal fashion. NTS has reciprocal direct or indirect projections to several higher CNS centers, including the parabrachial nucleus, hypothalamus, limbic system and forebrain providing the neuroanatomical substrate for the integration of various sensory, affective and emotional responses to nausea and vomiting (34).

CVS is viewed as a final common phenotype driven by synergistic interaction of discrete pathophysiological pathways. Similar to other periodic disorders, such as migraine, CVS might be characterized by a specific-individual “attack threshold” above which the synergistic action of the different pathophysiologic mechanisms induces the distinctive clinical expression. Each mechanism is not necessary pathogenetic, but it can be deemed as essential building unit within a common stimulus of adequate intensity able to breach the threshold for inducing the emetic cycles in susceptible patients (35). As the threshold may widely differ among patients, the development of effective and personalized treatments might rely on recognizing triggers and their underlying mechanisms and in turn either raising or desensitizing the individual threshold.

Several pathophysiologic mechanisms have been postulated, such as autonomic abnormalities, hypothalamic-pituitary-adrenal (HPA) activation, genetic abnormalities, neuronal hyperexcitability, and gastric dysmotility.

### Autonomic and Neuroendocrine Dysfunctions

Clinical manifestations of the autonomic nervous system (ANS) activation are dominant clinical features of CVS during both prodromal and acute phase. An increased sympathetic tone with low-to-normal parasympathetic tone during the interspersed period has been reported in both pediatric and adult CVS patients (36, 37). Postural orthostatic tachycardia syndrome (POTS) is diagnosed in up to 50% of the adolescents with CVS, and its treatment is effective in preventing emetic episodes (38, 39). The hypothalamus, which is functionally integrated into the limbic system, is considered the main ANS control center (33).

Stressors, both psychological (heightened emotional state) and physical (intercurrent infection, sleep deprivation, excessive exercise and prolonged fasting) can activate a neuroendocrine stress-mediated response by the HPA axis. Corticotropin-releasing factor (CRF), the major physiological activator of HPA axis and released from hypothalamic paraventricular nucleus (PVN), stimulates the release of ACTH and in turn cortisol from the adrenal cortex. However, CRF can also act in extra-hypothalamic circuits. Different types of CRF and different CRF receptors have been identified not only in CNS but also in the enteric nervous system. CRF-containing neurons from PVN project within NTS, where CRF receptors have been demonstrated as well as to the area postrema (40–42). Both central and peripheral injections of CRF inhibit gastric and proximal small bowel motor activity and induce vomiting in experimental animal and humans (43). Finally, it is also well known that NTS, via both catecholaminergic and non-catecholaminergic neurons, projects to the PVN regulating HPA axis and driving autonomic response to both acute and chronic stressors (44). Sato et al. (45) described a subset of children with CVS with prolonged and severe emetic phase associated with profound lethargy, hypertension and laboratory evidence of HPA axis hyper-responsiveness and increased secretion of antidiuretic hormone (ADH). Noteworthy, CRF exhibits a circadian rhythm, showing an increased secretion starting at 1 a.m. and reaching its peak at 6 p.m., which could account for the early morning onset of emetic phase.

CVS could be the consequence of a dysfunctional allostasis, defined as the physiologic adaptive changes activated by acute and chronic stressors for preserving the body homeostasis (46). Over time and with increasing stressor severity, the allostatic load may impair normal function leading to the development of pathology. The systems mediating allostasis include the HPA axis, ANS, metabolic systems, and the immune system. Hence, the hypothalamus plays a central role in orchestrating the physiological processes of stress adaptation. It has been suggested that early life negative events and negative life experiences might shape the development of neural circuits for cognitive and emotional processing and in turn, lead to disordered allostasis and decreased emetic “threshold” (35).

### Gastric Dysmotility

Gastric motor abnormalities have been suggested to play a key role in CVS pathogenesis. Chong et al. studied the gastric myoelectrical activity and gastric emptying time (GET) in 15 CVS children showing the presence of tachygastria in both preprandial and postprandial period and delayed gastric emptying (47). Conversely, Hejazi et al. (48) assessed GET using 4-h scintigraphic methods in 92 adults with CVS during the interspersed period of the disease and found rapid GET in 59% of patients, in 27% normal GET and in only 14% delayed GET, paralleling similar results previously reported in both adults and children (49, 50). It was postulated that rapid GET might reflect underlying autonomic dysfunctions reported in CVS patients; however, Hejazi et al. (48) failed to show any correlation between gastric emptying and autonomic testing results. Another hypothesis has speculated the role of ghrelin, a gut hormone able to enhance gastric emptying, in the pathogenesis of rapid GET during the remission period. Hejazi et al. (51) found increased ghrelin levels in adults with CVS compared with normal GET. However, the majority of the studies that have identified either rapid or normal GET were performed during the interspersed period, while those performed during the emetic phase have shown a significant gastric emptying delay, which might be related to either the activation of HPA axis resulting in the release of CRF, which inhibits foregut motility, or activation of dorsal vagal complex (DVC), which inhibits gastric motility via the efferent vagal pathway.

### Mitochondrial Dysfunction

The role of mitochondrial dysfunction in CVS pathophysiology was postulated based on the striking maternal inheritance pattern, the presence of an energy-depletion pattern on urine organic acid measurements and the efficacy of mitochondrial-targeted therapies, such as coenzyme Q10, L-carnitine, and riboflavin (52–54).

The genotype/phenotype correlation remains unclear as well as the functional role of mitochondrial dysfunction has yet to be determined. A simplistic underlying hypothesis is that mtDNA polymorphisms might impact energy metabolism during both a resting state, by decreasing the ability to preserve transmembrane ion gradients and hence predisposing to a hyperexcitability state, and during stress circumstances by failing to mount a greater energy supply for increased demand.

### Ion Channel Disease Abnormalities

Abnormalities in stress-induced calcium channel might also have a significant role in the CVS pathogenesis. Lee et al. found a significant association between the type 2 ryanodine receptor (RYR2), encoding a stress-induced calcium channel present in many central and peripheral neurons, and CVS [OR = 6.0, (95% CI =1.7–22)] (55).

### Neuronal Dys-Excitability Disorder

Neuronal hyperexcitability may be a common link between CVS and other episodic CNS disorders (11, 56, 57). Hyperexcitability may represent a consequence of genetic functional variants in mtDNA, ion channel and/or neurotransmitter receptor structure, or may result from aberrant neural circuits development. Alterations in brain network functional connectivity, particularly within networks involving the amygdala and the insular cortex, seem to play a role of brain “dysexcitability” in CVS patients (35).

### Endocannabinoid System Dysfunction

The cannabinoid receptor (CB) 1 and 2, their ligands N-arachidonoylethanolamine (anandamide) and 2- arachidonoylglycerol (2-AG), and their biosynthetic and degradative enzymes are the major components of the endocannabinoid system (ECS) (58). The ECS represents an important physiologic regulator of GI motility both centrally and peripherally. CB receptors are densely expressed in CNS areas, such are DVC, and in the enteric nervous system (59). The central inhibition of emetic reflex via CB1 receptor occurs by modulating vagal afferent activity within the DVC in the hindbrain, and vagal efferent activity projecting to enteric nervous system (60, 61). Venkatesan et al. (62) measured serum endocannabinoids and their related lipids, N-oleoylethanolamine (OEA) and N- palmitoylethanolamide (PEA), in 22 adults with CVS patients during both the acute emetic phase and the interspersed period, and 12 matched controls and found increased serum levels of endocannabinoid-related lipids during both phases.

### Toward a Unifying Hypothesis?

CVS may be best described as a consequence of dysfunction in the brain stem and hypothalamic nuclei that normally modulate or gate sensory emetic inputs, leading to the failure of brain integration and filtering mechanisms and resulting in the activation of emetic neurocircuitry under normal conditions. A mechanistic search for a common denominator focuses on the generalized central neuronal hyperexcitability, genetically driven by mutations in genes coding for ion-channels and mutations in mtDNA. Mitochondrial dysfunction impacts energy production at rest and fails to mount a greater energy supply during a period of heightened demand. Hence, common physical and psychological stressors might initiate the emetic cascade by stimulating dysfunctional hypothalamic neurons, characterized by high intrinsic energy demands, and consequently activating the autonomic nervous system and HPA axis with CRF release. The hypothalamus projects within NTS, which in turn activates the visceral and somatic motor pathways of the emetic cascade. Similarly, physical and psychological stressors might also initiate the emetic cascade directly activating NTS neurons, which by projecting to the PVN in the hypothalamus might stimulate both the HPA axis and autonomic responses.

## Clinical Manifestations

**Figure 1:**
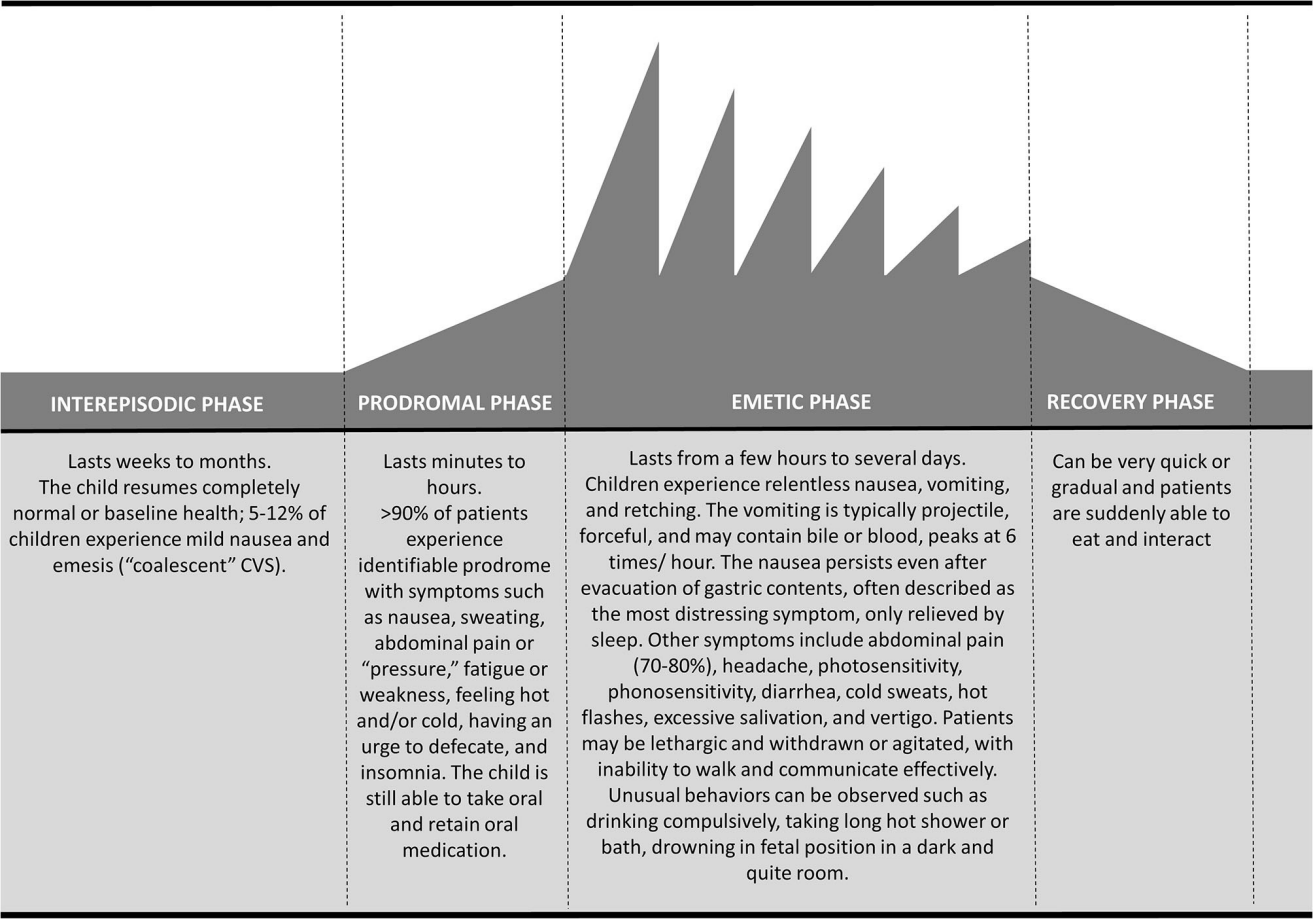
Temporal pattern of cyclic vomiting syndrome: schematic representation of the four phases.

CVS is characterized by stereotypical episodes of paroxysmal vomiting and intense unremitting nausea with a return to baseline health between episodes (1, 7, 8). This distinctive on-off temporal pattern characterized by four phases is essential for diagnosis (1, 8, 63) (Figure 1). Up to 75% of children exhibit symptoms during the night or early in the morning (generally 2.00–7.00 a.m.) (25, 64, 65) lasting several hours to days, although rarely >72 h (1). A study conducted on 181 children reported duration of attacks ranging from few hours to 10 days (mean 4.25 days) with intervals of 0.25–12 months (mean 1.8 months) (25).

Four phases have been identified: prodromal; emetic; recovery, and inter-episodic (63) (Figure 1). About 90% of patients experience a prodromal phase (63), that is characterized mainly by signs and symptoms of autonomic dysfunction such as pallor, sweating, lethargy, hot flashes and rarely temperature change and drooling (25, 65). It generally occurs a few hours before the vomit onset, and it might resemble a panic attack; this premonitory phase is similar to that of migraine headache attack (26). Abdominal pain is described in the prodromal as well as in the other phases (66). In approximately three-quarters of patients, recurrent stressors can be identified to precede CVS episodes. Emotional stress (generally of an excitatory nature) and infections are the most common triggers. Certain foods (e.g., chocolate, cheese, and caffeine), fasting, fever, lack of sleep, allergies, dietary and menstruation are also common trigger factors (20, 38, 67). The emetic phase is characterized by a projectile, intense vomit, averaging 6 times/hour at the peak (first hour), often leading to significant dehydration (1). Vomiting is often bilious (1) and associated to other gastrointestinal symptoms, such as abdominal pain, which is described in up to 80% of children, retching, anorexia, disabling nausea and diarrhea (68). Autonomic dysfunction can be exacerbated during this phase together with other neurological symptoms like headache, photophobia, phonophobia and vertigo (38, 68). Drowsiness and deep sleep are typical of the recovery phase; subsequently, children slowly start to re-tolerate food and beverages with remission of nausea and restoration of appetite (1). After the episode, children return to normal or baseline state of health lasting weeks to months (inter-episodic phase). Up to 12% of patients might experience interictal nausea and emesis episodes (“coalescent” CVS), usually less severe than those during a full episodes (8, 38).

## Diagnosis

There are three main different sets of criteria to consider for diagnosis of CVS in children (Table 1). The NASPGHAN (1), and the Rome IV (7, 8) classifications are those mainly used in the pediatric literature. The third classification was provided by ICHD (6), which in its 3rd edition (beta version) includes the CVS among the episodic syndromes potentially associated with migraine.

**Table 1:** Current classification for the diagnosis of pediatric Cyclic Vomiting Syndrome (CVS).

**NASPGHAN**
All of the criteria must be met
1. At least five attacks in any interval or a minimum of three attacks during a 6-months period
2.Episodic attacks of intense nausea and vomiting lasting 1 h to 10 days and occurring at least 1 week apart
3. Stereotypical pattern and symptoms in the individual patient
4.Vomiting during attacks occurs at least 4 times/h for at least 1 h
5. Return to baseline health between episodes
6. Not attributed to another disorder
**ROME IV**
**Children and Adolescents**
Must include all of the following
1.The occurrence of 2 or more periods of intense, unremitting nausea and paroxysmal vomiting, lasting hours to days within a 6-months period
2.Episodes are stereotypical in each patient
3. Episodes are separated by weeks to months with return to baseline health between episodes
4.After appropriate medical evaluation, the symptoms cannot be attributed to another condition
**NEONATES AND TODDLERS**
*Must include all of the following*
1.Two or more periods of unremitting paroxysmal vomiting with or without retching, lasting hours to days within a 6-months period
2.Episodes are stereotypical in each patient
3.Episodes are separated by weeks to months with return to baseline health between episodes of vomiting
**ICHD-3**
A.At least five attacks of intense nausea and vomiting, fulfilling criteria B and C
B.Stereotypical in the individual patient and recurring with predictable periodicity
C.All of the following:
1. nausea and vomiting occur at least four times per hour
2. attacks last ≥1 hour and up to 10 days
3.attacks occur ≥1 week apart
D.Complete freedom from symptoms between attacks
E.Not attributed to another disorder (In particular, history and physical examination do not show signs of gastrointestinal disease)

The key difference between classifications is represented by the number of recurrent episodes of vomiting required for formulating the diagnosis of CVS. Both NASPGHAN and ICHD guideline recommend a minimum of five attacks of intense nausea and vomiting for the diagnosis in children (1, 6), while a minimum of two episodes are required in Rome IV criteria (7, 8). The rationale behind this decision of the Rome IV working group was the possibility to make an early diagnosis of CVS. Moreover, compared to the other classifications, in Rome IV pediatric committee established different sets of criteria for neonates/toddlers (7) and children/adolescents (8). In the former set, the word “nausea” has been left out because of the difficulty in assessing in this symptom in infants (69).

Roma IV criteria recognize that some patients may not be completely asymptomatic in between typical episodes. Indeed, inter-episodic nausea, dyspepsia, and IBS symptoms might be experienced in 5–12% of children (38).

**Figure 2:**
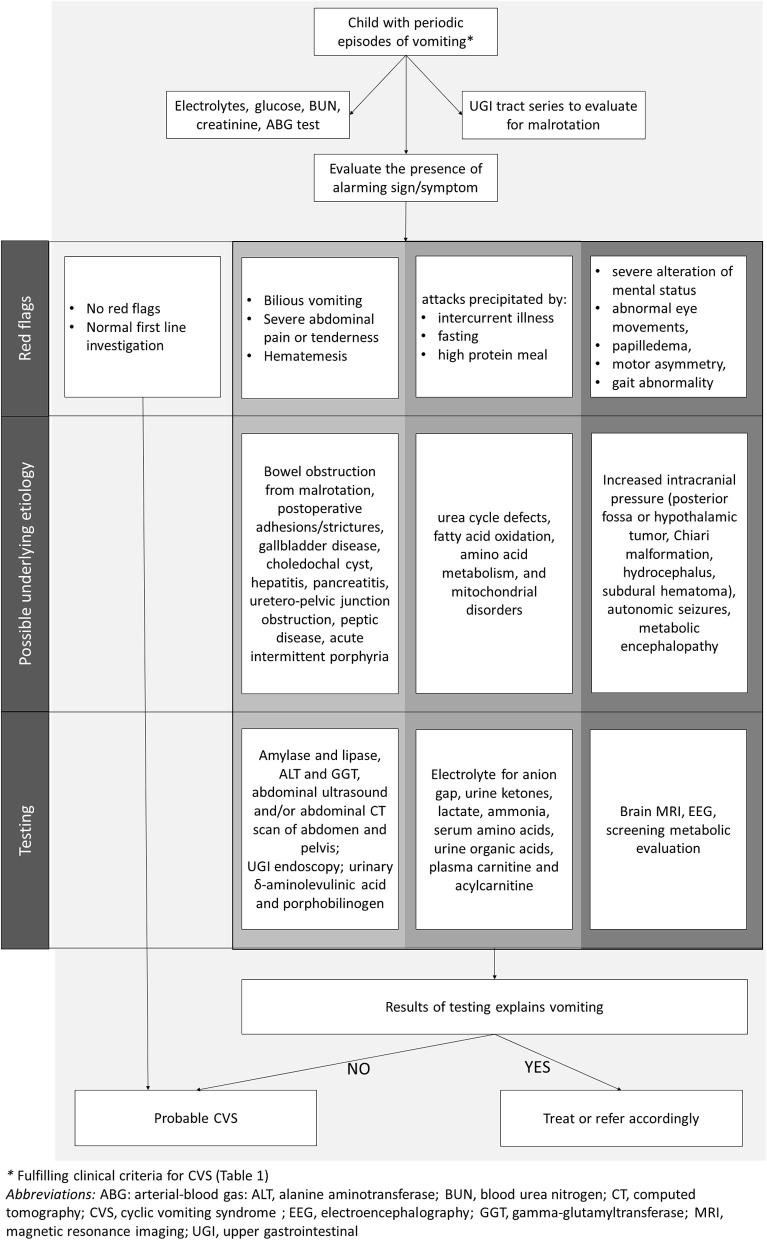
Evaluation of children with cyclic vomiting pattern.

A detailed medical history is a key to CVS diagnosis, so extensive and invasive investigations can be avoided. However, since serious metabolic, neurologic and surgical conditions may underlie the clinical picture of recurrent vomiting (1), it is recommended that all children should undergo baseline testing toward identifying organic causes (Figure 2). Screening includes basic metabolic profile (electrolytes, glucose, blood urea nitrogen, creatinine), to be performed before administration of intravenous fluids, and upper gastrointestinal tract series to exclude malrotation and anatomic obstructions (1, 7, 8). In children refractory to the initial treatment, transient hydronephrosis should be sought by abdominal ultrasound, preferably during a crisis. Addison disease and disorders of fatty acid oxidation should be excluded if a child has hyponatremia or hypoglycemia (1). An awake and/or sleep EEG should be performed to recognize autonomic seizures (Panayiotopoulos Syndrome) (70–72).

NASPGHAN guidelines indicate alarm symptoms and signs that may help clinicians in identifying those patients in whom further diagnostic testing is appropriate (1) (Table 2, Figure 2). In general, the occurrence of CVS under the age of 2 years raises the index of suspicion for neurometabolic diseases (1, 7, 8). When attacks are precipitated by acute illness, fasting or high-protein meals, metabolic and mitochondrial disorders need to be considered. Metabolic screening should be promptly performed for urea cycle defects, fatty acid oxidation, amino acid metabolism, and mitochondrial disorders.

**Table 2:** Clinical features suggestive of organic disorder.

**Alarm symptoms and signs**	**Additional testing**
Bilious vomiting, abdominal tenderness and/or severe abdominal pain	Amylase and lipase, alanine aminotransferase and g-glutamyltransferase; abdominal ultrasound and/or abdominal CT scan of abdomen and pelvis; upper GI endoscopy; urinary δ-aminolevulinic acid and porphobilinogen
Hematochezia ± melena	Upper GI endoscopy
Attacks precipitated by intercurrent illness, fasting, and/or high protein meal	Electrolyte for anion gap, urine ketones, lactate, ammonia, serum amino acids, urine organic acids, plasma carnitine and acylcarnitine
Abnormalities on neurological examination including severe alteration of mental status, abnormal eye movements, papilledema, motor asymmetry, and/or gait abnormality (ataxia)	Brain magnetic resonance imaging, electroencephalography, metabolic evaluation
History of head trauma	Brain magnetic resonance imaging
Progressively worsening episodes or conversion to a continuous or chronic pattern	Testing aimed at excluding chronic condition (e.g., inflammatory bowel disease) or metabolic disorders
Prolonged vomiting (>12 h in a neonate; >24 h in children; <2 years; >48 h in older children)	
Poor weight gain or weight loss	

Despite most children with CVS may experience bilious emesis and severe abdominal pain, they might also underline the presence of serious surgical and non-surgical disorders. Therefore, an investigation aimed at ruling out bowel obstruction from malrotation or postoperative complications, gallbladder disease, choledochal cyst, hepatitis, pancreatitis, or uretero-pelvic junction obstruction should be performed (Table 2). An upper GI endoscopy may be required if patients experience chronic gastrointestinal symptoms or large amounts of hematemesis. If anxiety, depression, hallucination, seizures, cranial nerve weakness, and paresis of the extremities are associated with vomiting and abdominal pain, detection of increased urinary δ-aminolevulinic acid and porphobilinogen in spot urine during the episode confirm the diagnosis of acute intermittent porphyria.

Adolescents should be questioned about the chronic marijuana use to identify a condition termed “cannabinoid hyperemesis syndrome” (CHS) which is characterized by severe cyclical nausea, vomiting, and abdominal pain that are relieved by compulsive long hot water bathing (1, 8, 72).

## Natural History/Prognosis

CVS resolves, in most children (50–70%) in late childhood or early adolescence (28–30, 73, 74). In one study among 41 children with CVS, 39% of children reported resolution of symptoms either immediately or within weeks from diagnosis. However, a large number of children from the group whose vomiting resolved continued to have somatic symptoms, with 42% of children suffering regular headaches and 37% having abdominal pain (compared to 50% of the persisting vomiting patients). Overall, 32% of the group had intermittent diarrhea and 54% experienced travel sickness at follow-up. Noteworthy, 78% of parents felt that the provision of a positive diagnosis and information made a significant impact on the severity of vomiting (29).

Resolution of symptoms did not correlate with duration or severity of the disorder at presentation or with any of the other variables analyzed (sex, age at diagnosis, admission to the hospital, identification of trigger factors, travel sickness, family history of migraine) (29).

According to another study on 28 cases (adult and children) with CVS, 62% of patients showed a gradual improvement in symptoms and 24% had complete resolution after a mean of 7 years (28).

Many children with CVS stop having emetic episodes as they grow older, although they develop headache throughout clinical history. Less commonly, CVS persists in adulthood or it may even begin in adulthood (75). Adult patients could be divided into subgroups with pediatric-onset (presentation before age 18) or adult-onset of CVS (37). A retrospective study (23) analyzed 101 CVS patients comparing those with pediatric-onset (29%) and those with adult-onset (71%). Pediatric-onset CVS patients were more likely to be female and there was a long delay in diagnosis when compared to adult-onset. Apart from these differences, both groups of patients had similar clinical characteristics and response to standard medications used in the treatment of CVS.

CVS is part of the episodic syndrome that may be associated with migraine (6). They are considered as an early life expression of migraine, thus they may occur without a headache component. A recent study on 1,134 children with tension-type headache (26.8%) or migraine (73.2%) found a previous history of “episodic syndromes” in 70.3% of patients (76). Among them, 6.6% of patients suffered from CVS. While some studies (5, 77) suggested that the episodic syndromes are exclusively associated with migraine, according to Tarantino et al. (76, 78) “migraine equivalents,” including CVS, show a similar prevalence in children with either migraine or tension-type headache.

The strict relationship between CVS and pediatric primary headaches is supported by a study (31) evaluating the prevalence of primary headache in children with a history of CVS and benign paroxysmal torticollis (BPT). The authors showed that 79% of patients with the previous history of CVS had developed headache (71% migraine and 29% tension-type headache).

## Comorbidities

CVS is probably not the result of a single pathogenetic mechanism, rather the common final clinical picture (cyclical emesis attacks) of different physiopathological pathways, with different threshold, and many triggers can be able to elicit it (79). Anxiety and mood symptoms affect about 59% of school-aged children with CVS and represent the most prevalent comorbidities (1, 80). Anxiety alone has been described in a quarter of CVS population (66); it may lead to school avoidance, worsening CVS-induced disability. Quality of Life has been demonstrated to be correlated with trait anxiety and coping abilities (80, 81).

Autonomic function in CVS has been extensively investigated since many of these patients exhibit autonomic dysregulation; abnormalities in skin sympathetic responses and thermoregulatory sweat tests have also been reported. Postural orthostatic tachycardia syndrome has been described in 14% to 38% of CVS adolescents (39), and many children with CVS showed evidence of altered autonomic tone at baseline with elevated sympathetic tone and low to normal parasympathetic tone (36). Chelimsky et al. (39) suggested that treating the underlying autonomic dysfunction reduces the number of vomiting episodes in CVS and many children can have a reduced number of vomiting episodes from fluid administrations, salt supplementation, fludrocortisone, and low-dose propranolol (82).

Among gastrointestinal comorbidities, a recent survey on an adult population showed that CVS was significantly associated with irritable bowel syndrome, gastroparesis, and gastroesophageal reflux. Pareek et al. (83) found that irritable bowel syndrome and/or a family history of irritable bowel syndrome were more commonly reported in CVS patients (67 vs. 62%) than in general population (10–20 vs. 14%).

Sleep hygiene and melatonin intake before bedtime to induce sleep onset may reduce the triggering effect of sleep deficit. Using frequent or longer-lasting energy sources (protein bars) and coenzyme Q10 (10 mg/kg/day), can improve stamina and participation in school and extracurricular activities (53).

CVS and migraine, both, can be triggered by acute psychological or physiological stress, sleep deprivation and menses (27, 35, 63, 84), and a personal or family history of migraine disorders are frequent in both children and adults with CVS.

Even epilepsy and panic disorder share some clinical features with CVS and it is important to stress that the autonomic manifestations are prominent (or even isolated) features in some epilepsy with onset in pediatric age such as PS and rolandic epilepsy, requiring sometimes a challenging differential diagnosis (70, 71). Additional neurologic findings such as developmental delay, seizures, hypotonia with or without neuromuscular disease manifestations, cognitive impairment, myopathy, and cranial nerve dysfunction have been reported in up to 29% of CVS patients allowing to propose a subtype called CVS plus (85).

## Differential Diagnosis

It includes seven main disorders that can be grouped in the acronym “URGENTIME”: URologic, Gastrointestinal, Endocrine, Neurologic disorders, Toxins/medications, (recurrent) Infections, and MEtabolic diseases. Specifically, renal colic and/or pelvic-ureteric junction obstruction may cause recurrent vomiting of urologic origin with possible symptom-free interval periods. Many gastrointestinal disorders may determine recurrent vomiting due to bowel obstruction (malrotation with volvulus, duplication cyst, and intermittent intestinal intussusception, chronic intestinal pseudo-obstruction), allergic or inflammatory process (food allergy, eosinophilic esophagitis, gastritis, duodenitis, hepatitis, biliary tract dysmotility, pancreatitis, pancreatic pseudocyst, appendicitis, peptic disease, inflammatory bowel disease); Pheochromocytoma, diabetes and Addison disease and different neurological disorders (epilepsy, migraine, autonomic nervous system disorders, brain tumor), should also be considered in children presenting with vomiting. Besides, toxins (such as the use of cannabis) and medications (antibiotics, NSAID, laxatives, hormones) need to be excluded. Also, recurrent infections, particularly enteritis, hepatitis, otitis media and chronic sinusitis may manifest with vomiting. Finally, several metabolic diseases such as aminoaciduria, organic aciduria, urea cycle and fatty acid oxidation defects, mitochondrial disorders and acute intermittent porphyria should be ruled out (86).

Because of the wide range of underlying conditions and lack of a specific sign and biomarker of CVS, the selection of first step and progression of investigations is often challenging and tests should be selected based on clinical presentation and suspicion (Tables 3–5).

**Table 3:** Diagnostic tests for ruling out conditions in the differential diagnosis with Cyclic Vomiting Syndrome (CVS).

**Condition**	**Diagnostic testing**
**GASTROINTESTINAL DISORDERS**	
Peptic ulcer disease	Upper GI endoscopy
Gastroparesis	Scintigraphic gastric emptying study
Hepatitis	Abdominal Ultrasound
Pancreatitis	Abdominal Ultrasound
Cholecystitis	Abdominal Ultrasound
Biliary tract anomalies	Hepatobiliary scintigraphy, endoscopic retrograde cholangiopancreatography, magnetic resonance cholangiopancreatography
Malrotation with volvulus, postoperative adhesions/strictures	Upper gastrointestinal series with small bowel follow through, abdominal CT scans, upper GI endoscopy
Chronic intestinal pseudo-obstruction	Plain abdominal X-ray, upper GI series with small bowel follow through, antroduodenal manometry
**EXTRA-INTESTINAL DISORDERS**	
Central nervous system	
Mass	Brain MRI, Brain CT
Hydrocephalus	Brain MRI, Brain CT
Subdural hematoma	Brain CT
Autonomic seizures	EEG
Renal Disorders	
Uretero-pelvic junction obstruction	Abdominal Ultrasound
Nephrolithiasis	Abdominal Ultrasound, abdominal CT

**Table 4:** Differential diagnosis between Cyclic Vomiting Syndrome and intracranial masses.

	**Cycling vomiting syndrome**	**Intracranial masses**
Prodromal symptoms	Always present	Rarely present
Dehydration	Always present	Rarely present
Warning signs^*^	Absent	Often present
MRI alterations	Absent	Present

* *Headache, altered sensory, papilledema, hypertension, bradycardia or tachycardia, signs of herniation, retinal hemorrhages, bluish skin lesions, fractures, ataxia, cranial nerve deficits, motor/sensory deficit, seizures, visual dysfunction*.

**Table 5:** Relevant causes of vomiting in metabolic disorders.

**ASSOCIATED OR NOT WITH ENCEPHALOPATHY**
Organic acidurias
Urea cycle disorders
Fatty acid oxidation disorders
MCT1 defect
MELAS
Glutaric aciduria type I
**ASSOCIATED WITH ACIDOSIS/KETOACIDOSIS**
Organic acidurias
Mitochondrial diseases
**ASSOCIATED WITH KETOSIS ONLY**
Ketolysis defects
**ASSOCIATED WITH SEVERE ABDOMINAL PAIN**
Phorphyrias (acute intermittent porphyria, coproporphyria)
**ASSOCIATED WITH HEPATOPATHY**
Organic acidurias
Urea cycle disorders
Galactosemia
Hereditary fructose intolerance
Tyrosinemia type I
Fatty acid oxidation disorders

### Neurological Disorders

#### Epilepsy

Vomiting may be an “ictal” manifestation, as a part of the seizure semeiology (87, 88), and in young children, autonomic phenomena such as nausea and vomiting are common symptoms of PS, an age-related childhood-onset focal idiopathic epilepsy. PS is often misdiagnosed as encephalitis, migraine, gastroenteritis, gastroesophageal reflux or CVS (70, 71, 89). Carbonari et al. showed that CVS is a common misdiagnosis in children with PS and other non-convulsive epilepsies (90). Other epilepsies such as temporal lobe epilepsy (TLE) or symptomatic epilepsies related to posterior regions of the brain can manifest with vomiting as the main manifestation and mimic CVS (91).

#### Migraine

As CVS and migraine share common pathogenic mechanisms (39), many clinical features of CVS, as well as a family history of migraine, are in common with migraine; moreover, patients with CVS often manifest migraine later in life.

Abdominal pain is one of the key symptoms of cyclic vomit and it is also the major feature of abdominal migraine, moreover, abdominal migraine and CVS can co-exist in the same child (13). Abdominal migraine is a migraine subtype where children have attacks presenting predominantly with abdominal, rather than headache symptoms. The International Classification of Headache Disorders defines abdominal migraine as recurrent attacks of moderate to severe midline abdominal pain lasting 2–72 h, associated with flushing, pallor, anorexia, nausea, or vomiting without headache. At least five episodes are needed to fulfill the diagnosis. Children are normal between attacks and gastrointestinal or renal disorders are ruled out (6–8). Despite all the confusing overlaps, in abdominal migraine pain predominate over vomiting, while nausea and vomiting predominate over abdominal pain in CVS (92); also, certain pain characteristics are more likely in CVS such as burning, non-midline, mild and not interfering in daily activities, and duration of >1 h (13).

#### Autonomic Nervous System Disorders

Many of the symptoms of CVS that are associated with episodes of vomiting such as pallor, increased salivation, nausea, abdominal pain and unwillingness have been attributed to autonomic imbalance (39, 82). Thus, CVS should be distinguished from dysautonomic disorders, such as acute autonomic neuropathy (93) and hereditary sensory or autonomic peripheral neuropathies.

#### Cannabinoid Hyperemesis Syndrome (CHS)

This condition is characterized by paroxysmal episodes of abdominal pain, nausea and vomiting in individuals addicted to daily cannabis or marijuana use. Screening for cannabinoid use has to be considered in adolescents with unexplained CVS (91). Often, individuals suffering from CHS report temporary cessation of symptoms after hot bathing and showers, a helpful clue to differentiate CHS from CVS (92).

#### Brain Tumors and Other Intracranial Masses

Brain tumors and other intracranial masses (hydrocephalus, posterior fossa tumors, subdural hematoma, and subdural effusion) represent a differential diagnosis of cyclic vomiting.

They can cause nausea, vomiting, or both, by increasing the intracranial pressure (ICP) at the area postrema of the medulla. Vomiting, often present in the morning, occurs due to increasing of ICP during the night while the patient is sleeping and venous drainage is decreased (32). Acute elevation of ICP needs timely treatment, so it's important to look for some warning signs such as headache, altered sensory, papilledema, hypertension, bradycardia or tachycardia, signs of herniation, retinal hemorrhages, bluish skin lesions and fractures.

While CVS is characterized by stereotypical episodes with prodromal symptoms such as nausea, abdominal pain, anorexia and pallor, emesis in brain tumor or other intracranial masses occur without prodromal symptoms and is triggered by a rapid change in body position and nausea is rarely present (15). Brain tumors and strokes may present with focal neurologic deficits and the type is depending by location and disease stage (Table 4).

Another differential diagnosis is idiopathic intracranial hypertension (pseudotumor cerebri) (94). In these patients, ICP is increased with normal cerebrospinal fluid (CSF) content, normal neuroimaging and absence of other neurological signs. It mostly affects obese adolescent girls and is typically described by other authors with headache and sometimes nausea and vomiting. In summary, intracranial expansive masses are often associated with neurological findings, including ataxia, cranial nerve deficits, motor/sensory deficit, seizures, visual dysfunction, papilledema, so it is important never to ignore pediatric patients with these symptoms.

### Metabolic Diseases

Recurrent vomiting is a characteristic clinical sign of inborn errors of metabolism (IEMs), such as organic acidurias (usually in association with acidosis), disorders of urea cycle (with hyperammonemia) and fatty acid oxidation defects (Table 5). Laboratory investigation for metabolic causes of vomiting should include glucose, ketonemia, acid-base balance, lactate, ammonia, acylcarnitine and urinary organic acid. The association of other clinical and biochemical abnormalities may direct the differential diagnosis.

#### Vomiting With Encephalopathy

Chronic or recurrent vomiting in infancy is particularly common to the organic acidurias (OAs) due to a defect in the metabolism of branched-chain amino acids isoleucine, leucine and valine, in which the accumulation of small molecules proximal to the metabolic block, which are toxic for the body, especially for the brain and are therefore defined intoxication-type IEMs (95). Neurological damage is characteristic with associated symptoms ranging from poor feeding to slow growth, lethargy, vomiting, dehydration, malnutrition, hypoglycemia, hypotonia, metabolic acidosis, ketoacidosis, and hyperammonemia.

Vomiting may be due to the hyperammonemia associated with disorders of the urea cycle (UCDs), inborn errors of ammonia detoxification/arginine synthesis (96). In severe cases, a rapid deterioration of the level of consciousness can be observed, but, in milder affected patients, vomiting can be the only presenting symptom and/or be intermittent. Fatty acid oxidation disorders can also manifest with hyperammonemia and vomiting, because of metabolic decompensation due to prolonged fasting or infections. As recurrent vomiting leads to alkalosis, the evidence of acidosis at acid-base analysis should raise the suspicion of an OAs or other causes of loss of bases; conversely, vomiting in UCDs is associated with alkalosis. Along with recurrent metabolic vomiting, some patients with OA and UCDs present with focal neurological signs or cerebral edema. These patients can be mistakenly diagnosed as having brain tumors or cerebrovascular accidents. Another rare organic aciduria, Glutaric Aciduria type I. frequently presents with encephalopathic episodes and vomiting, mimicking encephalitis, in association with an intercurrent gastrointestinal or viral infection. This disorder is caused by an inherited deficiency of glutaryl-CoA dehydrogenase, which is involved in the catabolic pathways of L-lysine, L-hydroxylysine and L-tryptophan (97). Prompt recognition of this disorder permits the start of a low lysine diet and carnitine supplementation, improving neurological outcome (98).

Noteworthy, in several countries, these conditions are included in the panels of Expanded Newborn Screening, allowing earlier diagnosis and treatment.

#### Cyclic Vomiting With Severe Abdominal Pain

The Porphyrias are IEMs due to defect of the biosynthesis of heme, which enters in the composition of cytochromes as well as hemoglobin. Diffuse crampy abdominal pain and constipation are present in all the three most common acute intermittent porphyrias, variegate and hereditary coproporphyria (99). Acute neurovisceral symptoms are due to increased activity of the first step of porphyrin synthesis and can be aggravated by certain drugs. The abdominal pain may be intense, similarly to that which occur in diabetic ketoacidosis. Striking accumulations and excess excretion of heme pathway intermediates and their oxidized products give a characteristic red (or dark) urine color. Hepatic porphyrias are transmitted as an autosomal dominant trait. Diagnosis is often difficult and a positive urine screening test (Watson –Schwarts test) may be present only during acute illness. Concomitant study of blood, urine, and stool for porphyrins is the best diagnostic approach, followed by genetic analysis (99).

#### Vomiting With Ketosis

While ketonuria should always be considered abnormal in neonates, it is a physiological result of catabolism in late infancy, childhood, and even adolescence. However, hyperketosis >6 mmoles/l of total plasma ketone bodies that cause metabolic acidosis (serum bicarbonate <18 mmol/l) is always pathological. Ketosis in absence of other biochemical abnormalities such as acidosis, hyperlactatemia, or hypoglycemia, rarely is due to an IEMs and is likely to be a normal physiological response to fasting, catabolism, vomiting, medium-chain triglyceride enriched or other ketogenic diets). Conversely, ketoacidosis with or without hypoglycemia could be seen in several metabolic disorders, especially OAs and mitochondrial diseases.

Persistent ketosis (both in fasting and in fed state) suggests a genetic defect of ketolysis. This category includes deficiency of Beta-Ketothiolase, due to mutation of ACAT1 gene, and defect of succynil-CoA:3 Oxoacid CoA transferase, caused by pathogenic variants in SCOT gene (100). Both disorders are characterized by acute episodes di nausea and vomiting, often leading to encephalopathy and coma. Metabolic studies show in both disorders an increase of 3OH-butyrate in serum and urine. Beta-Ketothiolase is also associated with a characteristic profile of acylcarnitine and urinary organic acids.

Monocarboxylate transporter type 1 deficiency (MCT1) is caused by mutations in the *MCT1* gene (*SLC16A1*) on chromosome 1p13. MCT1 has been reported as a cause of recurrent episodes of severe ketoacidosis often associated with cycling vomiting without consciousness depression (101).

#### Vomiting With Hepatopathy

Galactosemia, Hereditary Fructose Intolerance (HFI) and Tyrosinaemia type I, are the main conditions in this category characterized by vomiting plus acute liver failure, requiring immediate and specific treatment (102). Patients may show acute deterioration, vomiting, seizures, dehydration, hypoglycaemia, liver failure and tubulopathy. Other biochemical abnormalities associated with liver disease are mellituria, hyperammonaemia, hyperlactatemia, hypoglycaemia, hypertyrosinaemia, and hypermethioninaemia. The presentation of Tyrosinaemia type I is usually after the 3rd week of life, whereas galactosaemia usually presents in the newborn period and HFI after weaning, since fructose is not normally part of infant formulas. In the suspect of one of these conditions, galactose, fructose and proteins must be excluded from the diet, pending confirmation of the diagnosis. When galactosaemia or HFI is confirmed, proteins can be reintroduced (102). If Tyrosinaemia type I is confirmed, patients should start immediate treatment with NTBC, along with a low-phenylalanine and low-tyrosine diet, to help a rapid recovery from acute liver failure (103).

#### Differential Diagnosis With Cyclic Vomiting of Childhood

Cyclic vomiting of childhood, often triggered by fasting and in the setting of infection, needs to be differentiated from IEMs. In some children, episodes can also be provoked by intense exercise. Typically, episodes begin in the second year and usually end within puberty. Urinary organic acid analyses show prominent ketosis, but no pathological metabolites, and acylcarnitine analysis shows prominent acetylcarnitine. Treatment with intravenous glucose usually results in rapid resolution of the symptoms. Ondasentron can be effective, whereas phenotazine antiemetic is of limited use. A substantial number of children with this phenotype cannot be included in a precise disease category (104). Some authors have suggested an impaired uptake of ketone bodies into the peripheral tissues. This disorder could be sometime confused with ketotic hypoglycemia, but blood glucose is not abnormally low, and the treatment for ketotic hypoglycemia (avoiding fasting, cornstarch at bedtime, etc.) is not particularly beneficial (105).

## Genetics Findings in CVS

CVS running through generations has been sporadically reported (106–109). Moreover, inherited inborn errors of metabolism, including fatty acid oxidation disorders, urea cycle defects mitochondrial and amino acids disorders, have been associated with pediatric CVS (4, 55, 110–114). However, so far, CVS has an entry [MIM # 500007] in the online catalog of Mendelian Inheritance in Man (115), currently attributed in this catalog to mutations in the mitochondrial transfer RNA-leucine [*MTTL1*; MIM # 590050] gene (107). Mitochondrial dysfunction [i.e., mitochondrial DNA polymorphisms [including A3243G, C16519T, and G3010A mtDNA polymorphisms and mutations in the *MTTL1* mitochondrial gene (MIM # 590050) or mitochondrial DNA rearrangements or deletions] has been demonstrated in some patients (4, 53, 67, 91, 107–109, 111–113, 116–121). The functional significance of these single nucleotide polymorphisms remains unknown. Furthermore, these mitochondrial associations have not been replicated in adults with cyclic vomiting (112) suggesting the role of other non-mitochondrial factors (11).

Individuals with CVS may also harbor (polymorphic) mutations in single genes, including: (1) *RYR2* (ryanodine receptor 2) [MIM # 180902; on chromosome 1q43] (2) (55) *SCN4A* (sodium channel voltage-gated, type IV subunit alpha) [MIM # 603967; on chromosome 17q23.3] (3) (11) CNR1 (cannabinoid receptor 1) [MIM # 114610; on chromosome 6q15] (122); and (4) OPRM1 (opioid receptor MU1) [MIM # 600018; on chromosome 6q25.2] (122).

The *RYR2* gene encodes for a stress-induced calcium release channel receptor two, which is part of the ryanodine receptor [RYR: a tetramer composed of 4 RYR2 polypeptides and four FK506-binding proteins or FKBP12.6], present in the sarcoplasmic reticulum of (a) cardiac muscular cells [where it acts as the major source of calcium, required for cardiac muscle excitation-contraction coupling], being responsible for (type 2) right ventricular dysplasia with cardiac arrhythmia type 2 [MIM # 600996] and (type 1) catecholaminergic polymorphous ventricular tachycardia [MIM # 604772]; and (b) autonomic and other neurons. Marx et al. (123) demonstrated that protein kinase A [PKA; MIM # 176911; on chromosome 7p22.3] phosphorylation of RYR2 dissociates FKBP12.6 and regulates the channel open probability: in defective hearts RYR2 is PKA hyperphosphorylated, resulting in defective channel function due to increased sensitivity to calcium-induced activation.

The *SCN4A* gene encodes for a component (i.e., the alpha subunit four) of the voltage-gated sodium channel integral membrane protein, which form a pore in the cytoplasmic membrane conducting sodium ions through the membrane and is responsible, so far of: (a) a group of related muscular disorders, including hyperkalemic periodic paralysis [HYPP; MIM # 170500], paramyotonia congenita [PMC; MIM # 168300]; and (b) a group of disorders classified as potassium-aggravated myotonia [MIM # 608390], and hypokalemic periodic paralysis type 2 [HOKPP2; MIM # 613345].

The *CNR1* gene encodes for the G-protein coupled pre-synaptic cannabinoid receptor 1 (**CB**_**1**_), which is expressed in glutamatergic and GABAergic interneurons, located prevalently in the central (e.g., hippocampus, basal ganglia, cerebellum, neocortex, and spine), peripheral and autonomic (e.g., heart and gut) nervous system but also in the endocrine and sexual glands, and is activated by endocannabinoids acting as a modulator, which in turn decreases the release of glutamate and GABA. Interestingly, the use of cannabinoids is relatively common among individuals with CVS and chronic cannabis use has been associated paradoxically with cannabinoid hyperemesis syndrome (124–126).

The *OPRM1* gene encodes for the primary site of action for the endogenous enkephalins and beta-endorphins, and is located in the pre- and post-synaptic regions of neurons distributed over the brain (e.g., periacqueductal region, dorsal horns of the spine, olfactory bulb, and neocortex) and intestinal tract.

An unifying genetic-related pathogenic mechanism infers that the synergic roles of these nuclear DNA mutations and single-gene sequence variants may result in aberrant stress-induced calcium release [RYR2-mediated] into the mitochondria (11), or sodium release/balance [SNN4A-mediated] across the cytoplasmic membrane, or neurotransmitter modulation [e.g., CNR1- or OPRM1-mediated] or axonal transport (KIF1B) or energy production (TRAP1) of autonomic neurons, resulting in an increased risk to develop autonomic/functional disease such as cyclic vomiting, and related conditions such as migraine, epilepsy and gut dysmotility: this model incorporates the existing hypotheses regarding CVS pathogenesis into a cohesive mechanism, and might have treatment implications (52, 127).

Chew and al. (128) expanded a “TUBB3 E410K phenotype” originally known as type 3 congenital fibrosis of the extra-ocular muscles [CFEOM; MIM # 600638; on chromosome 16q24.3] due to mutations in *TUBB3* [tubulin beta-3; MIM # 602661], which encodes for the two heterodimer proteins that compose microtubules, by adding, besides congenital fibrosis of the extra-ocular muscles, facial weakness, developmental delay and progressive sensorimotor peripheral neuropathy, Kallmann syndrome [i.e., hypogonadotropic hypogonadism and anosmia; MIM # 308700], stereotyped midface hypoplasia, intellectual disabilities and, in some cases, vocal cord paralysis, tracheomalacia and cyclic vomiting. They inferred that the c.1228G > A mutation in the *TUBB3* gene and subsequent E410K amino acid substitution in the beta-tubulin 3 protein, defines a new genetic etiology for Moebius syndrome [MIM # 157900], Kallmann syndrome and cyclic vomiting (128).

Larger genetic and functional studies of CVS in both adults and children will be needed to better establish the heritable basis of this disorder. These studies must involve well-characterized patient sub-sets to better delineate genotype-phenotype relationships.

## Focus on Emergency Department

Evaluation and recognition of etiology of pediatric patients with recurrent vomiting in the emergency department (ED) can represent a challenge because a broad differential diagnosis is present. While the most common causes are benign, it can be due to potentially disabling or life-threatening conditions, requiring early diagnosis, and prompt intervention. Misdiagnosis is not uncommon (129) with consequent ED discharge with a non-specific diagnosis and consequent diagnostic delay, quantified in 2.5 years (38). It has been described that patients may have up to 10 ED visits per year, with over 5 ED visits before the correct diagnosis (130). The emergency physician should use a systematic approach that has to be age and developmentally appropriate to be able to identify life-threatening emergencies, such as bowel obstruction, diabetic ketoacidosis, adrenal crisis, toxic ingestion, or increased intracranial pressure (131). Diagnosing CVS efficiently and cost-effectively can be achieved by early clinical recognition based upon clinical criteria (Table 1), followed by a limited diagnostic workup to exclude alternative disorders in all patients, even in the absence of warning signs for organic causes (132) (Figure 2).

In ED setting biochemical, radiographic assessments, and potentially endoscopic/ultrasound assessment should be considered. Biochemical testing should include arterial-blood gas test, complete blood count, serum electrolytes and glucose, liver panel, lipase, blood urea nitrogen, creatinine and urinalysis, also to exclude possible dehydration (132). So especially in urgent care, the physicians should: (1) investigate for the etiology of vomiting, taking into account the child's age (serious disorders are more frequent in children younger than 2 years); (2) look for the possible consequences or complications of vomiting (e.g., fluid depletion, hypokalemia, and metabolic alkalosis) and correct it; (3) provide a targeted therapy, when possible or, in other cases, the symptoms should be treated (131). The NASPGHAN Consensus on pediatric CVS proposed in 2008 a diagnostic workup (1), in which especially during the acute phase, the tendency toward an “exclusion” diagnosis prevails through the execution of first level investigations. A careful anamnestic collection and physical examination can orientate toward the identification of those children with warning signs/symptoms in which level II investigations are necessary (Table 6).

**Table 6:** Pediatric emergency department protocol for patients with recurrent vomiting regardless of an established diagnosis of Cyclic Vomiting Syndrome (CVS).

**Medical history and physical examination**
- individuate the presence of warning signs/symptoms (Table 2)
- evaluate for the presence of clinical criteria for CVS (Table 1)
- evaluate the change in the typical vomiting pattern (if previous CVS diagnosis)
- evaluate previously performed investigations
**Emergency Department management**
1. Clinical assessment: pulse rate, temperature, breathing rate, blood pressure, level of consciousness, state of hydration, and body weight
2. Investigations for all patients:
• complete blood count, electrolytes, glucose, blood urea nitrogen, creatinine, arterial-blood gas test, urinalysis
• upper gastrointestinal tract series to evaluate for malrotation (if not done before)
3.Other laboratories and diagnostic imaging guided by warning symptoms/signs at discretion of attending physician:
• Attack with bilious vomiting, severe abdominal pain, abdomen tenderness, hematemesis
∘ *At any time*
▪ ultrasound of abdomen and pelvis, lipase/amylase, EGDS
∘ *During the attack*
▪ Amylase and lipase, alanine aminotransferase and g-glutamyltransferase
▪ Upper GI endoscopy if large hematemesis
**• Attack precipitated by fasting, intercurrent illness, high protein meal**
∘ *Before IV fluid*
▪ glucose, electrolytes for anion gap, lactate, pyruvate, ammonia, serum amino acid, urine organic acid, urine ketones, plasma carnitine, acylcarnitine
**• Abnormal neurological exam (severe altered mental status, abnormal eye movement, papilledema, motor asymmetry, gait abnormality)**
▪ Brain MRI, Brain CT
▪ EEG
4.Reassess after treatment for emetic phase of CVS (see treatment) or for complications of vomiting
• *Treatment failure*: intensify treatment as indicated (see treatment) or admit patient
• *Positive treatment response*: discharge with treatment advice and eventually referral to specialist

*This ED protocol represents a sample template and should be tailored based on individual needs*.

It is recommended that in the presence of red flag further evaluation is performed. Once life-threatening causes are excluded and acute alterations are treated, the patient should be referred to an appropriate pediatric specialist (e.g., gastroenterologist, surgeon, neurologist, metabolic experts), to avoid inappropriate further ED visits and a delay in the diagnosis and preventive therapy in case of CVS (130).

Further evaluation:

1. If the acute onset of unilateral or flank pain, severe abdominal pain, abdominal tenderness bilious vomiting, perform ALT/GGT (LFT's), lipase ± amylase, an abdominal and pelvic ultrasound to exclude e.g., gallstones or acute hydronephrosis should be performed (132). Large amounts of upper gastrointestinal bleeding may warrant endoscopic evaluation. Persisting upper gastrointestinal symptoms between episodes suggest performing an upper endoscopy at any time between episodes to rule out other causes (e.g., coeliac disease, IBD). In case of suspected obstructive disorders (e.g., bilious vomiting, severe discomfort) in addition to LFTs, lipase, ultrasound, also a plain abdominal X-ray or CT abdomen should be performed.
2. If metabolic warning, blood and urine tests should be obtained, followed by delivery of 10% dextrose-containing intravenous fluid at a rate of 1.5 times maintenance (simultaneously with fluid boluses as necessary) (1). During the early part of the episode (before IV fluids are administered) physicians should measure: serum concentrations of lactate, pyruvate, ammonia and serum amino acids, blood gas in addition to serum electrolytes (for anion gap) as well as urine organic acids (in addition to urine ketones already done); eventually carnitine, acylcarnitine and urine catecholamines, δ-aminolevulinic acid and porphobilinogen should be carried out especially in patients with supporting symptoms (e.g., anxiety, depression, hallucination, seizures, cranial nerve weakness, and paresis of the extremities). After obtaining the appropriate specimens for testing (before IV fluids are administered), emergency treatment must be instituted. If it not possible to carried out these tests in the ED or to save a small amount of frozen urine and plasma for later evaluation. Moreover, an upper gastrointestinal series to the ligament of Treitz (a small bowel follow-through or CT/MR enterography) should be performed in all children to exclude malrotation or non-fixation with possible intermittent volvulus.
3. If neurologic signs, perform magnetic resonance imaging (MRI) of the brain or computed tomography (CT) to rule out intracranial lesions, brain tumors or referral to a neurologist. Moreover, in those patients with neurologic signs an electroencephalography (EEG) recording should also be performed to rule out PS, a benign epileptic syndrome, characterized by predominantly autonomic symptoms (including emesis): the availability of EEG recording in pediatric ED might be useful for a prompt and not-cost-consuming diagnosis (133).
4. Children who present in ED with a CVS diagnosis (Rome IV criteria) and without any additional warning symptoms require only a limited set of further investigation. During each episode, laboratory testing should be performed, consisting of electrolytes, glucose, blood urea nitrogen (BUN), creatinine, and urinalysis to primary monitor for acute hypovolemia and electrolyte disturbances. It is clear, e.g., that mild metabolic acidosis, hypoglycemia, and ketosis are consistent with CVS while severe acidosis or hypoglycemia (in particular non-ketotic hypoglycemia) warrant further evaluation for an inborn error of metabolism, especially in infants and toddlers. It is also important to identify triggers (in particular infections), and recognize comorbid (132).

## Treatment

The management of CVS requires an individually tailored therapy that takes into consideration the frequency and severity of attacks, and resultant disability balanced against the potential side effects of treatment. The two key treatment arms are prophylactic measures and medications administered in the interictal period and acute and supportive interventions given during attacks (1) (Table 7).

**Table 7:** Medications available for pharmacological treatment of Cyclic Vomiting Syndrome (CVS) in children.

**Medications**	**Class**	**Mechanism of action**	**Goal and indication**	**Dose**	**Route of administration**	**Side effects**
Sumatriptan	Antimigraine	5HT1B/1D agonist	Prodromal phase as abortive therapy	10 mg <40 Kg 20 mg > 40 kg (age × 4 + 20)/100 × 3 mg, in children 12 years and older	Intranasal subcutaneous	Neck pain/burning and coronary vasospasm and it is contraindicated in basilar artery migraine
Ondansetron	Antiemetic	5-HT3 receptor antagonist	Abortive therapy	0.3-−0.4 mg/kg/dose every 4–6 h, max 20 mg/day)0.15 mg/kg per dose recommended.	Intravenous oral/sublingual in patients with milder symptomatology	constipation, dry mouth, headache, drowsiness, QT prolongation
Cyproheptadine	Antimigraine	Anti-histamine, serotonin (5HT2) and calcium channel antagonist	Preventative First choice in children ≤ 5 years	0.25–0.5 mg/kg/day Single night-time dose or divided bid or tid.	Oral	Increased appetite, weight gain and sedation
Pizotifen	Antimigraine	Serotonin (5HT2) antagonist and anti-histamine	Preventative alternative to cyproheptadine Available only in Canada and the UK	0.5–1.5 mg at night	Oral	increased appetite, weight gain and sedation
Propranolol	Antimigraine	β-blockers	Preventative	0.25–1 mg/kg/day, most often 10 mg bid or tid	Oral	lethargy, reduced exercise tolerance, bradycardia
Erythromycin	Antiemetic	Prokinetic agent	Preventative	20 mg/Kg/day	Oral	
Aprepitant	Antiemetic	Neurokinin (NK1) receptor antagonist	Preventative Phase as abortive therapy	40 mg orally twice/week in children **<** 40 kg, 80 mg in children 40–60 kg, and 125 mg in children **>** 60 kg 125 mg 30 min before the emetic phase, followed 80 mg/day 2–3 >20 kg, 80 mg for 3 days 15- 20 kg, 80 mg/day 40 mg day 2–3 <15 kg.	Oral	hiccups, fatigue, increased appetite, mild headache and severe migraine
Amitriptyline	Antidepressant	Tricyclic antidepressant	Preventative	Starting dose should be 0.2–0.3 mg/kg/day and increases of 5–10 mg/week should be done up to the highest dose of 1–1.5 mg/kg/day	Oral	Dry mouth, constipation, weight gain, morning tiredness, behavioral changes, cardiotoxicity (tachyarrhythmia)
Phenobarbital Valproic acid Topiramate	Anticonvulsants	Barbiturate either multiple mechanism of action	Preventative	2–3 mg/kg/day at bedtime 10–40 mg/kg/day 2 mg/kg/day divided in 2 daily doses	Oral	Sedation, cognitive impairment, Hyperactivity, disruptive behavior Irritability, Anorexia/weight loss, Hypertermia/dehydratation
Flunarizine	Antimigraine	Non-selective calcium channel blocker	Preventative	5 mg per day	Oral	Hypotension, Weight gain and appetite
Fluoxetine	Antidepressant	Selective serotonin reuptake inhibitor	Preventative	20 mg/day as anxiolytic treatment (not enough evidence)	Oral	Gastrointestinal symptoms, Sleep changes, Headaches. Restless legs. Appetite changes
Carnitine		Mitochondrial supplements	Alternate preventive	50–100 mg/kg/day, adults 1 g tid	Oral	Diarrhea, fishy body odor
Co-enzyme Q10		Mitochondrial supplements	Alternate preventive	5–10 mg/kg/day, adults 100 mg tid	Oral	Diarrhea
Riboflavin		Mitochondrial supplements	Alternate preventive	400 mg daily or divided twice daily	Oral	Not described
Ketorolac	Analgesic	Non-steroidal anti-inflammatory	Supportive	0.4–1 mg/kg per dose every 6 h, max dose 30 mg, max daily dose 120 mg	Intravenous	gastrointestinal bleeding and dyspepsia
Omeprazole	Decreases stomach acid production	Proton pump inhibitors	Supportive	0.1 mg/kg	Intravenous	
Lorazepam	Sedatives	5-HT3 receptor antagonist	Supportive as rescue therapy	0.05–0.1 mg/kg/dose iv every 6 h, max 4 mg.	Intravenous	Disorientation, dizziness, hypotension, respiratory depression
Chlorpromazine	Sedatives, antiemetic, antipsychotic	D_2_-antagonist	Supportive as rescue therapy	0.5–1 mg/kg/dose every 6 h, max 40 mg/day <5 years; max 75 mg/day 5–12 years	Intravenous	Drowsiness, hypotension, seizure, extrapyramidal symptoms, arrhythmias
Diphenhydramine (only in association with chlorpromazine)	Sedatives, antiemetic, antihistamine	H_1_-antagonist	Supportive as rescue therapy	1–1.25 mg/kg/dose every 6 h	Intravenous	Respiratory depression, hallucinations, hypotension, nausea, blurred vision

### Acute Treatment

Therapeutic management of acute phase is mainly based on supportive and on symptomatic care aimed to correct fluid and electrolyte deficits, provide antiemetic therapy, analgesics, and sedation for relief of unrelenting nausea, vomiting, and pain. Moreover, early intervention with abortive agents during the brief prodromal phase can be used to attempt to terminate the attack (79, 134).

Sumatriptan (5HT_1B/1D_agonist) can be used intranasally (10 mg <40 Kg−20 mg >40 kg) or subcutaneous [(age × 4 + 20)/100 × 3 mg] in children 12 years and older (127, 135). It has also been shown that it is more effective when there is a family history of migraines. Uncommon side effects include neck pain/burning and coronary vasospasm and it is contraindicated in basilar artery migraine (134, 135).

Aprepitant, a neurokinin (NK1) receptor antagonist, can be used during the prodromal phase as abortive therapy. In a retrospective study, 25 pediatric patients refractory to conventional CVS therapies were treated with aprepitant at the beginning of the prodromal phase (Table 7), resulting in a decrease in vomiting duration and frequency in 76% of the patients (136). Once the vomiting starts, patients should be admitted to the hospital to provide supportive care and interventions aimed to stop the emetic phase (79).

Supportive care includes: (1) decrease stimulation in a dark, quiet, private room with minimum vital sign measures; (2) replacement of fluids, electrolytes and energy balance. It has been reported that the use of 10% dextrose solutions is associated with an improvement of the catabolic state and of ketosis that could exacerbate nausea (134). Vomiting can lead to hypokalemia and potassium replacement might be necessary. In case of prolonged fasting with minimal energy and/or protein intake, temporary nasojejunal feedings or parenteral nutrition can hasten recovery (1, 79) Treatment of pain and complications. Ketorolac (0.4–1 mg/kg per dose intravenously every 6 h, max dose 30 mg, max daily dose 120 mg) is considered the first-line analgesic treatment for pain. In selected severe cases, morphine or fentanyl can be used (134). The association with intravenous H2-receptor antagonist or proton pump inhibitors at conventional dosage can be helpful to treat epigastric pain and also to prevent esophagitis and hematemesis from Mallory-Weiss tear (1). Transient hypertension found in the SATO subset of CVS should be treated with short-acting ACE inhibitors (e.g., captopril) during the episode only. If secretion of the antidiuretic hormone with hyponatremia, low serum osmolality, and high urine specific gravity occurs, water intake should be restricted until values normalize (1). Metabolic acidosis can occur for several causes and should be checked taking arterial blood gas and treated if needed (134). Ondansetron iv (0.3–0.4 mg/kg/dose every 4–6 h, max 20 mg/day) has been shown to decrease vomiting duration or frequency during the acute phase by more than 50% (38). It can be used at a dose of 0.15 mg/kg per dose oral/sublingual in those patients with milder symptomatology; main side effects are constipation, dry mouth, headache, drowsiness (20). Moreover, since QT prolongation can occur with the administration of this medication, a baseline ECG is recommended.

When ondansetron fails to control nausea and vomiting, sleep induced by sedatives may be the only way to provide symptomatic relief. The most effective combination is ondansetron and lorazepam (0.05–0.1 mg/kg/dose iv every 6 h). Alternatively, chlorpromazine (0.5–1 mg/kg/dose every 6 h) and diphenhydramine (1–1.25 mg/kg/dose every 6 h) can be used together, but this provides less antiemetic and more sedative effect (38). In extreme cases, dexmedetomidine has been successfully used to treat three pediatric CVS patients by a continuous infusion in the intensive care setting (137).

If a child does not respond to one of the discussed regimens or the episode differs from previous ones by greater severity, longer duration, or different symptoms, then the clinician should consider the possibility of an underlying disease (e.g., acute appendicitis, pancreatitis, brain tumor) and the need for new or to repeat diagnostic testing (e.g., abdominal ultrasound, brain TC/MRI) (79).

The recovery phase from the last emesis to the successful retention of food and drink typically lasts a few hours. Once children want to eat food, they can generally return to a normal diet without gradual progression. However, some children experience protracted symptoms including intractable nausea with the inability to eat, persistent dizziness and hyperesthesia with allodynia; antiemetics, proton pump inhibitors or anticholinergic agents, and analgesics respectively are of little help (79).

### Prophylactic Treatment and Lifestyle Changes and Dietary Restrictions

During the interictal period, lifestyle changes and the use of reassurance (e.g., attacks are not self-induced) and anticipatory guidance (e.g., improvement with age and knowledge that effective therapies are available) can themselves have a significant therapeutic effect reducing the frequency of attacks (138). In patients with anxiety, cognitive behavioral therapy and biofeedback may be needed (137).

A careful history and a detailed vomiting diary recording frequency of episodes, type of meal before episodes, and potentially aggravating life events can help to identify and avoid potential triggers in 70% of children (38). For these reasons, a short-term trial of 1–2 months to assess the impact of these conservative measures may be established synchronized with the diagnostic workup aimed to exclude organic causes of vomiting.

Lifestyle changes include: (1) avoidance of excessive excitement (e.g., birthdays, holidays, and overexertion); (2) avoidance of triggering foods. Although extensive dietary restriction of potential triggering foods is not recommended, it is reasonable to test eliminating foods or chemical substances that appear to be aggravating factors for migraines (e.g., cheese, chocolate, hot dogs, aspartame, monosodium glutamate. and alcohol) (139). Also, children with documented food sensitivities to specific foods (e.g., cow, soy, or egg white proteins) have been shown to improve following specific dietary elimination (3) (140) Consumption of high-carbohydrate snacks between meals, before physical exertion, and at bedtime should be used when a patient's history suggests fasting-induced attacks (38). Furthermore, given that CVS is considered to be within the migraine spectrum, it is appropriate to suggest migraine lifestyle interventions that include good sleep hygiene (e.g., regular sleep schedules, avoidance of sleepovers), regular aerobic exercise, regular meal schedules, maintenance of good hydration, and moderation or avoidance of caffeine (141). Finally, marijuana consumption should be checked in adolescents because its use was found to worsen the cyclic hyperemesis and its cessation decreased episodes of vomiting (142).

The indication for prophylactic pharmacotherapy depends on attack intensity (more than every 1–2 months) and severity (exceeding 2 days or requiring hospitalization), the impairment of the QoL (e.g., frequent school absences) and if attack treatments are ineffective or cause side effects. The choice of prophylactic pharmacological treatment should take into account the age of the child, psychological comorbidities and formulation and safety profile of the drug. The low initial dose is recommended, and increase incrementally, titrating to effect (1).

Cyproheptadine is a first-generation antihistamine used in GI disorders for its serotonin (5HT2) and calcium channel antagonist effects (143, 144). Cyproheptadine is effective in young children with CVS and is the first choice for children 5 years old or younger (145–147). The recommended dose is 0.25–0.5 mg/kg/day divided twice or three times per day. Common side effects include increased appetite, weight gain and sedation. Increased weight due to enhanced appetite makes this drug the best choice in an underweight patient but not recommended in school-age girls. To reduce the sedation experienced during the school day it can be successfully used as a single night-time dose (147).

Pizotifen 0.5–1.5 mg at night is an alternative to cyproheptadine with the same side effects. However, it is available only in Canada and the United Kingdom (148).

Propranolol is a β-blockers recommended as the second choice in children of all ages (1). Haghighat et al. (25) showed in a randomized trial that propranolol was effective in 74 out of 83 (92%) patients and appeared to be more effective than amitriptyline). Interestingly, the addition of a daily oral dose of erythromycin 20 mg/Kg to propranolol showed a significant increase in response rate in a randomized trial (149, 150).

Recommended propranolol dose is 0.25–1 mg/kg/day, most often 10 mg twice or three times per day. Main side effects are lethargy and reduced exercise intolerance, moreover, the resting heart rate should be monitored for potential bradycardia and when discontinued, it should be tapered for 1–2 weeks. It is contraindicated in patients with asthma, diabetes, heart disease and depression.

Only one uncontrolled study evaluates erythromycin alone (20 mg/kg/day) as a prokinetic agent but the strength of this data is limited by the poor quality of the study (151).

An interesting approach when standard agents are either ineffective or poorly tolerated is the use of aprepitant. Aprepitant was approved for the prevention of chemotherapy-induced nausea and vomiting (152, 153) (see Table 7 for aprepitant dose); at 12 months follow-up, 13 children (81%) achieved either complete (3/16, 19%), or partial (10/16, 62%) clinical response. Adverse effects to aprepitant included hiccups, fatigue, increased appetite, mild headache, and severe migraine; only one patient stopped the medication for severe migraines (135).

Amitriptyline, a tricyclic antidepressant, is the most widely prescribed prophylactic medication for the treatment of CVS (52). North American Society for Pediatric Gastroenterology, Hepatology and Nutrition suggests amitriptyline as the first-line treatment in children older than ≥5 years (1). In literature, several studies are supporting the efficacy of amitriptyline in large pediatric case series (23, 25, 27, 30, 145, 154). Badihian et al. (147) found that amitriptyline and cyproheptadine showed the same efficacy in CVS prophylaxis. A retrospective study (52) reported a similar level of efficacy for amitriptyline and CoQ-10. Bagherian et al. (155) showed that amitriptyline is a better choice to reduce the severity of CVS attacks compared to topiramate. Response rates of amitriptyline in the available case series range from 70 to 90 per cent (52, 147). Amitriptyline should be started at a low dose and slowly titrated up to the desired effect if tolerated. Starting dose should be 0.2–0.3 mg/kg/day and increases of 5–10 mg/week should be done up to the highest dose of 1–1.5 mg/kg/day (79, 156). Since the most common amitriptyline side effect is represented by sleepiness, the drug should be administered at bedtime. Amitriptyline can have also anticholinergic, arrhythmogenic, and behavioral side effects, thus it can cause constipation, dry mouth, sedation, QT prolongation, increased appetite. Although half of the children experience at least one side effect, only 19% have to stop the drug (52).

If a patient does not respond to the first-line therapy (amitriptyline, cyproheptadine, and/or propranolol) the following options should be considered: (1) presence of persisting triggers (e.g., psychological stressors) and comorbid conditions (e.g., anxiety, POTS) or missed underlying disorders (e.g., hydronephrosis, chronic sinusitis, acute appendicitis, intestinal malrotation with volvulus, CNS tumors, metabolic crises) and toxic exposure (e.g., cannabis); (2) inadequate compliance which is common in adolescents and can be documented by testing blood levels for amitriptyline; (3) response to specific medications in CVS is quite variable and often requires serial medication trials and dose escalation before efficacy is achieved; (4) use of combination therapy with 2 drugs (e.g., amitriptyline with propranolol or an anticonvulsant); (5) use of complementary therapy such as carnitine, coenzyme Q, estrogens, acupuncture, or psychotherapy.

The use of antiepileptics in CVS prophylaxis has been studied in a few clinical trials (30, 155, 157–160) (Table 7). Valproate (10–40 mg/kg/day) is effective for the prophylaxis of severe CVS (30, 159). Sezer and Sezer (160) compared topiramate with propranolol as a long-term treatment option. The responder rates were 81% for the propranolol group and 94% for the topiramate group. Topiramate should be started with 25 mg at night for 1 week, then increased in 25 mg increments at weekly intervals at the usual dose 50–100 mg per day in two divided doses (max. 200 mg per day) until to clinical control.

Flunarizine is a non-selective calcium channel blocker commonly used as a prophylactic treatment for episodic migraine (75, 161–163). The recommended dose in migraine prophylaxis is 5–10 mg daily. Common side effects include increased appetite, weight gain and sedation. Its use in CVS patients is supported by anecdotal cases.

The use of mitochondrial supplements (co-enzyme Q10, L-carnitine, and riboflavin) may be helpful in a subset of patients with suspected mitochondrial or metabolic dysfunction (53, 54, 164). Boles et al. (53) suggested that a protocol consisting of mitochondrial-targeted cofactors (co-enzyme Q10 and L-carnitine) plus amitriptyline (or possibly cyproheptadine in preschoolers) is highly effective and safe in the prevention of vomiting episodes. The dose of drugs used in CVS is summarized in Table 7.

Low estrogen oral contraceptives can be used to treat girls with menstrual-related CVS (165). Anecdotal experience suggests that acupuncture may attenuate the severity of CVS attacks (166). Psychotherapy, especially stress reduction, may help as adjunctive therapy (167, 168).

## Conclusion

In the first description of CVS, Dr. Samuel Gee in 1882 wrote: “These cases seem to be all of the same kind, their characteristic being fits of vomiting, which recurs after intervals of uncertain length. The intervals themselves are free from signs of disease.” His observations were later included in the definition of “the periodic syndrome of childhood” described by Wyllie and Schlesinger in 1933. CVS is now typified by stereotyped intense bouts of vomiting, at least 4 times per hour, lasting for hours to days followed by stretches of wellness. Although the recognition of CVS has been facilitated by the recently defined diagnostic criteria, many patients are still misdiagnosed. Moreover, at present, there are no specific tests for diagnosing CVS. Therefore, CVS is currently classified as an idiopathic disorder and the diagnosis relies on fulfilling clinical criteria.

CVS pathophysiology is still not well-understood; however, given the link between migraine and cyclic vomiting, it is assumed that there are similarities in the underlying cause. Over the last years, there have been some advancements in understanding the etiology and pathogenesis of CVS. However, CVS is currently still classified as an idiopathic disorder. Indeed, enlightening the pathophysiological mechanisms could unfold intriguing aspects of the syndrome, such as its periodicity, the mechanisms of actions of emetic triggers, and the heterogeneity in symptom severity and treatment response despite the phenotypic similarity.

There are no known ways to prevent or mitigate the risk in those with cyclic vomiting syndrome. The inheritance pattern is partial and there are no clear predictive markers of the disorder. If a child presents for a first or second episode of severe vomiting and there is a strong family history of migraine it might raise cyclic vomiting syndrome higher on the differential list and allow for earlier identification. Moreover, as CVS is a relatively uncommon condition there are no therapeutic controlled or open trials in the management of CVS and treatment recommendations are mainly based on expert opinion. Further clinical studies are crucial to assessing the efficiency and safety of the different treatment options and how the quality of life, the attack-free interval and the acute phase of the disease change with the antiepileptic drug compared to standard therapy.

## Data Availability Statement

The original contributions presented in the study are included in the article/supplementary material, further inquiries can be directed to the corresponding author/s.

## Author Contributions

UR, OB, GDN, RT, SSal, PS, and PPar conceived and planned this project inviting all the experts in this field from both the Italian Society of Pediatric Gastroenterology Hepatology and Nutrition (SIGENP) and Italian Society of Pediatric Neurology (SINP), they have also written the first draft of the manuscript. All the other involved authors participated in writing and improving, according to their specific expertise and experience, the final version of the manuscript.

## Conflict of Interest

The authors declare that the research was conducted in the absence of any commercial or financial relationships that could be construed as a potential conflict of interest.

## References

[1] Li BU Lefevre F Chelimsky GG Boles RG Nelson SP Lewis DW. North American society for pediatric gastroenterology, hepatology, and nutrition consensus statement on the diagnosis and management of cyclic vomiting syndrome. J Pediatr Gastroenterol Nutr. (2008) 47:379–3. 10.1097/MPG.0b013e318173ed39 18728540

[2] Heberden W Commentaries on the History and Causes of Diseases, 3rd ed London, UK: Payne and Foss, 1806 [cited by Hammond J. The late sequelae of recurrent vomiting of childhood. Dev Med Child Neurol. (1974) 16:15–22. 10.1111/j.1469-8749.1974.tb02706.x 4813486

[3] Gee S On fitful or recurrent vomiting. St. Bartholomew's Hospital Rep. (1882) 18:1–6.

[4] Zaki EA Freilinger T Klopstock T Baldwin EE Heisner KR Adams K. Two common mitochondrial DNA polymorphisms are highly associated with migraine headache and cyclic vomiting syndrome. Cephalalgia. (2009) 29:719–28. 10.1111/j.1468-2982.2008.01793.x 19220304

[5] Spiri D Rinaldi VE Titomanlio L. Pediatric migraine and episodic syndromes that may be associated with migraine. Ital J Pediatr. (2014) 40:92. 10.1186/s13052-014-0092-4 25928129 PMC4239406

[6] Headache Classification Committee of the International Headache Society (IHS) The International Classification of Headache Disorders, 3rd edition (beta version). Cephalalgia. (2013) 33:629–808. 10.1177/0333102413485658 23771276

[7] Benninga MA Faure C Hyman PE St James Roberts I Schechter NL Nurko S. Childhood functional gastrointestinal disorders: neonate/toddler. Gastroenterology. (2016) 130:1519–26. 10.1053/j.gastro.2005.11.065 27144631

[8] Hyams JS Di Lorenzo C Saps M Shulman RJ Staiano A van Tilburg M. Functional disorders: children and adolescents. Gastroenterology. (2016) 150:1456–68. 10.1053/j.gastro.2016.02.015 27144632

[9] Doi.Brezin F Wiedemann A Feillet F Cyclic vomiting syndrome in children. Arch Pediatr. (2017) 24:1129–36. 10.1016/j.arcped.2017.08.010 28947248

[10] Drossman DA Hasler WL. Rome IV-Functional GI disorders: disorders of gut-brain interaction. Gastroenterology. (2016) 150:1257–61. 10.1053/j.gastro.2016.03.035 27147121

[11] Hasler WL Levinthal DJ Tarbell SE Adams KA Li BUK Issenman RM. Cyclic vomiting syndrome: pathophysiology, comorbidities, and future research directions. Neurogastroenterol Motil. (2019) 31(Suppl. 2):e13607. 10.1111/nmo.13607 31241816 PMC6899706

[12] Sagar RC Sood R Gracie DJ Gold MJ To N Law GR. Cyclic vomiting syndrome is a prevalent and under-recognized condition in the gastroenterology outpatient clinic. Neurogastroenterol Motil. (2018) 30. 10.1111/nmo.13174 28745840

[13] Abu-Arafeh I Russell G. Cyclical vomiting syndrome in children: a population-based study. J Pediatr Gastroenterol Nutr. (1995) 21:454–8. 10.1097/00005176-199511000-00014 8583299

[14] Ertekin V Selimoglu MA Altnkaynak S. Prevalence of cyclic vomiting syndrome in a sample of Turkish school children in an urban area. J Clin Gastroenterol. (2006) 40:896–8. 10.1097/01.mcg.0000212627.83746.0b 17063107

[15] Fitzpatrick E Bourke B Drumm B Rowland M. The incidence of cyclic vomiting syndrome in children: population-based study. Am J Gastroenterol. (2008) 103:991–6. 10.1111/j.1572-0241.2007.01668.x 18070235

[16] Chogle A Velasco-Benitez CA Koppen IJ Moreno JE Ramírez Hernández CR Saps M A population-based study on the epidemiology of functional gastrointestinal disorders in young children. J Pediatr. (2016) 179:139–43.e1. 10.1016/j.jpeds.2016.08.095 27726867

[17] Prakash C Clouse RE. Cyclic vomiting syndrome in adults: clinical features and response to tricyclic antidepressants. Am J Gastroenterol. (1999) 94:2855–60. 10.1111/j.1572-0241.1999.01428.x 10520833

[18] Prakash C Staiano A Rothbaum RJ Clouse RE. Similarities in cyclic vomiting syndrome across age groups. Am J Gastroenterol. (2001) 96:684–8. 10.1111/j.1572-0241.2001.03606.x 11280534

[19] Aziz I Palsson OS Whitehead WE Sperber AD Simrén M Törnblom H. Epidemiology, clinical characteristics, and associations for Rome IV functional nausea and vomiting disorders in adults. Clin Gastroenterol Hepatol. (2019) 17:878–86. 10.1016/j.cgh.2018.05.020 29857155

[20] Kovacic K Sood M Venkatesan T. Cyclic vomiting syndrome in children and adults: what is new in 2018? Curr Gastroenterol Rep. (2018) 20:46. 10.1007/s11894-018-0654-5 30159612

[21] Li BU Murray RD Heitlinger LA Robbins JL Hayes JR. Heterogeneity of diagnoses presenting as cyclic vomiting. Pediatrics. (1998) 102(3 Pt 1):583–7. 10.1542/peds.102.3.583 9738180

[22] Stewart WF Lipton RB Liberman J. Variation in migraine prevalence by race. Neurology. (1996) 47:52–9. 10.1212/WNL.47.1.52 8710124

[23] Kumar N Bashar Q Reddy N Sengupta J Ananthakrishnan A Schroeder A. Cyclic Vomiting Syndrome (CVS): is there a difference based on onset of symptoms – pediatric versus adult? BMC Gastroenterol. (2012) 12:52. 10.1186/1471-230X-12-52 22639867 PMC3443054

[24] Bhandari S Venkatesan T. Clinical characteristics, comorbidities and hospital outcomes in hospitalizations with cyclic vomiting syndrome: a nationwide analysis. Dig Dis Sci. (2017) 62:2035–44. 10.1007/s10620-016-4432-7 28050780

[25] Haghighat M Rafie SM Dehghani SM Fallahi GH Nejabat M. Cyclic vomiting syndrome in children: experience with 181 cases from southern Iran. World J Gastroenterol. (2007) 13:1833–6. 10.3748/wjg.v13.i12.1833 17465476 PMC4149962

[26] Bhandari S Jha P Thakur A Kar A Gerdes H Venkatesan T. Cyclic vomiting syndrome: epidemiology, diagnosis, and treatment. Clin Auton Res. (2018) 28:203–9. 10.1007/s10286-018-0506-2 29442203

[27] Li BU Murray RD Heitlinger LA Robbins JL Hayes JR. Is cyclic vomiting syndrome related to migraine? J Pediatr. (1999) 134:567–72. 10.1016/S0022-3476(99)70242-8 10228291

[28] Lee LY Abbott L Moodie S Anderson S. Cyclic vomiting syndrome in 28 patients: demographics, features and outcomes. Eur J Gastroenterol Hepatol. (2012) 24:939–43. 10.1097/MEG.0b013e328354fc83 22617361

[29] Fitzpatrick E Bourke B Drumm B Rowland M. Outcome for children with cyclical vomiting syndrome. Arch Dis Child. (2007) 92:1001–4. 10.1136/adc.2007.116608 17588965 PMC2083597

[30] Hikita T Kodama H Ogita K Kaneko S Nakamoto N Mimaki M. Cyclic vomiting syndrome in infants and children: a clinical follow-up study. Pediatr Neurol. (2016) 57:29–33. 10.1016/j.pediatrneurol.2016.01.001 26861170

[31] Moavero R Papetti L Bernucci MC Cenci C Ferilli MAN Sforza G. Cyclic vomiting syndrome and benign paroxysmal torticollis are associated with a high risk of developing primary headache: a longitudinal study. Cephalalgia. (2019) 39:1236–40. 10.1177/0333102419844542 30982347

[32] Yang HR. Recent concepts on cyclic vomiting syndrome in children. J Neurogastroenterol Motil. (2010) 16:139–47. 10.1038/nm0210-139b 20535344 PMC2879837

[33] Hornby PJ. Central neurocircuitry associated with emesis. Am J Med. (2001) 111(Suppl. 8A):S106–12. 10.1016/S0002-9343(01)00849-X 11749934

[34] Sanger GJ Andrews PLR. Treatment of nausea and vomiting: gaps in our knowledge. Autonom Neurosci. (2006) 129:3–16. 10.1353/art.2006.0012 16934536

[35] Levinthal DJ. The cyclic vomiting syndrome threshold: a framework for understanding pathogenesis and predicting successful treatments. Clin Transl Gastroenterol. (2016) 7:e198. 10.1038/ctg.2016.55 27787513 PMC5288589

[36] To J Issenman RM Kamath MV. Evaluation of neurocardiac signals in pediatric patients with cyclic vomiting syndrome through power spectral analysis of heart rate variability. J Pediatr. (1999) 135:363–6. 10.1016/s0022-3476(99)70135-6 10484804

[37] Venkatesan T Prieto T Barboi A Li B Schroeder A Hogan W. Autonomic nerve function in adults with cyclic vomiting syndrome: a prospective study. Neurogastroenterol Motil. (2010) 22:1303–7, e339. 10.1111/j.1365-2982.2010.01577.x 20667005

[38] Li BU Balint J. Cyclic vomiting syndrome: evolution in our understanding of a brain-gut disorder. Adv Pediatr. (2000) 47:117–60. 10.1179/amb.2000.47.2.117 10959442

[39] Chelimsky G Madan S Alshekhlee A Heller E McNeeley K Chelimsky T. A comparison of dysautonomias comorbid with cyclic vomiting syndrome and with migraine. Gastroenterol Res Pract. (2009) 2009:701019. 10.1155/2009/701019 20111731 PMC2810453

[40] Miller AD Leslie RA. The area postrema and vomiting. Front Neuroendocrinol. (1994) 15:301–20. 10.1006/frne.1994.1012 7895890

[41] Taché Y. Cyclic vomiting syndrome: the corticotrophin releasing factor hypothesis. Dig Dis Sci. (1999). 44:S79–86. 10.1023/A:1026602216846 10490044

[42] Taché Y Bonaz B. Corticotropin-releasing factor receptors and stress-related alterations of gut motor function. J Clin Invest. (2007) 117:33–40. 10.1172/JCI30085 17200704 PMC1716215

[43] Taché Y Perdue MH. Role of peripheral CRF signalling pathways in stress-related alterations of gut motility and mucosal function. Neurogastroenterol Motil. (2004) 16 (Suppl 1):137–42. 10.1111/j.1743-3150.2004.00490.x 15066020

[44] Herman JP. Regulation of hypothalamo-pituitary-adrenocortical responses to stressors by the nucleus of the solitary tract/dorsal vagal complex. Cell Mol Neurobiol. (2018). 38:25–35. 10.1007/s10571-017-0543-8 28895001 PMC5918341

[45] Sato T Igarashi N Minami S Okabe T Hashimoto H Hasui M. Recurrent attacks of vomiting, hypertension and psychotic depression: a syndrome of periodic catecholamine and prostaglandin discharge. Acta Endocrinol. (1988) 117:189–97. 10.1530/acta.0.1170189 2837885

[46] McEwen BS. Stress, adaptation, and disease. Allostasis and allostatic load. Ann N Y Acad Sci. (1998) 840:33–44. 10.1111/j.1749-6632.1998.tb09546.x 9629234

[47] Chon GSK Electrogastrography in cyclic vomiting syndrome. Dig Dis Sci. (1999) 44(8 Suppl.):S64–73. 10490042

[48] Hejazi RA Lavenbarg TH Pasnoor M Dimachkie M Foran P Herbelin L. Autonomic nerve function in adult patients with cyclic vomiting syndrome. Neurogastroenterol Motil. (2011) 23:439–43. 10.1111/j.1365-2982.2011.01679.x 21323793

[49] Turchetti A Guglielmi S Fossati C Matrunola M Corrado G. Gastric emptying time in cyclic vomiting syndrome in children. Eur Rev Med Pharmacol Sci. (2004) 8:295–8. 15745390

[50] Fajardo NR Cremonini F Talley NJ. Frontiers in functional dyspepsia. Curr Gastroenterol Rep. (2005) 7:289–96. 10.1007/s11894-005-0021-1 16042912

[51] Hejazi RA Lavenbarg TH McCallum RW. Spectrum of gastric emptying patterns in adult patients with cyclic vomiting syndrome. Neurogastroenterol Motil. (2010) 22:1298–302, e338. 10.1111/j.1365-2982.2010.01584.x 20723071

[52] Boles RG Lovett-Barr MR Preston A Li BU Adams K. Treatment of cyclic vomiting syndrome with co-enzyme Q10 and amitriptyline, a retrospective study. BMC Neurol. (2010) 10:10. 10.1186/1471-2377-10-10 20109231 PMC2825193

[53] Boles RG. High degree of efficacy in the treatment of cyclic vomiting syndrome with combined co-enzyme Q10, L-carnitine and amitriptyline: a case series. BMC Neurol. (2011) 11:102. 10.1186/1471-2377-11-102 21846334 PMC3163531

[54] van Calcar SC Harding CO Wolff JA. L-carnitine administration reduces number of episodes in cyclic vomiting syndrome. Clin Pediatr. (2002) 41:171–4. 10.1177/000992280204100307 11999680

[55] Lee J Wong SA Li BU Boles RG. NextGen nuclear DNA sequencing in cyclic vomiting syndrome reveals a significant association with the stress-induced calcium channel (RYR2). Neurogastroenterol Motil. (2015) 27:990–6. 10.1111/nmo.12575 25925909

[56] Aanpreung P Vajaradul C. Cyclic vomiting syndrome in Thai children. J Med Assoc Thai. (2002) 85:S743–8. 12403255

[57] Shearer J Luthra P Ford AC. Cyclic vomiting syndrome: a case series and review of the literature. Frontline Gastroenterol. (2018) 9:2–9. 10.1136/flgastro-2016–100705 29484154 10.1136/flgastro-2016-100705 PMC5824764

[58] Pesce M D'Alessandro A Borrelli O Gigli S Seguella L Cuomo R. Endocannabinoid-related compounds in gastrointestinal diseases. J Cell Mol Med. (2018) 22:706–15. 10.1111/jcmm.13359 28990365 PMC5783846

[59] Howlett AC Barth F Bonner TI Cabral G Casellas P Devane WA. International Union of Pharmacology. XXVII. Classification of cannabinoid receptors. Pharmacol Rev. (2002) 54:161–202. 10.1124/pr.54.2.161 12037135

[60] Darmani NA Johnson JC. Central and peripheral mechanisms contribute to the antiemetic actions of delta-9-tetrahydrocannabinol against 5-hydroxytryptophan-induced emesis. Eur J Pharmacol. (2004) 488:201–12. 10.1016/j.ejphar.2004.02.018 15044052

[61] Izzo AA Sharkey KA. Cannabinoids and the gut: new developments and emerging concepts. Pharmacol Ther. (2010) 126:21–38. 10.1016/j.pharmthera.2009.12.005 20117132

[62] Venkatesan T Zadvornova Y Raff H Hillard CJ. Endocannabinoid related lipids are increased during an episode of cyclic vomiting syndrome. Neurogastroenterol Motil. (2016) 28:1409–18. 10.1111/nmo.12843 27098832 PMC5002231

[63] Fleisher DR Gornowicz B Adams K Burch R Feldman EJ. Cyclic vomiting syndrome in 41 adults: the illness, the patients, and problems of management. BMC Med. (2005) 3:20. 10.1186/1741-7015-3-20 16368014 PMC1326207

[64] Rashed H Abell TL Familoni BO Cardoso S. Autonomic function in cyclic vomiting syndrome and classic migraine. Dig Dis Sci. (1999) 44 (8 Suppl.):s74–8. 10490043

[65] Lindley KJ Andrews PL. Pathogenesis and treatment of cyclical vomiting. J Pediatr Gastroenterol Nutr. (2005) 41 (Suppl. 1):S38–40. 10.1097/01.scs.0000180299.04731.cb 16131963

[66] Redon S Mareau C Guedj E Donnet A. Cyclic vomiting syndrome in adults and children: a hypothesis. Headache. (2017) 57:943–51. 10.1111/head.13108 28488756

[67] Moses J Keilman A Worley S Radhakrishnan K Rothner AD Parikh S. Approach to the diagnosis and treatment of cyclic vomiting syndrome: a large single-center experience with 106 patients. Pediatr Neurol. (2014) 50:569–73. 10.1016/j.pediatrneurol.2014.02.009 24842256

[68] Abell TL Adams KA Boles RG Bousvaros A Chong SK Fleisher DR. Cyclic vomiting syndrome in adults. Neurogastroenterol Motil. (2018) 20:269–84. 10.1111/j.1365-2982.2008.01113.x 18371009

[69] Zeevenhooven J Koppen I Benninga M. The new Rome IV criteria for functional gastrointestinal disorders in infants and toddlers. Pediatr Gastroenterol Hepatol Nutr. (2017) 20:1–13. 10.5223/pghn.2017.20.1.1 28401050 PMC5385301

[70] Parisi P Pacchiarotti C Ferretti A Bianchi S Paolino MC Barreto M. Gastroesophageal reflux disease vs. panayiotopoulos syndrome: an underestimated misdiagnosis in pediatric age? Epilepsy Behav. (2014) 41:6–10. 10.1016/j.yebeh.2014.08.137 25269687

[71] Graziosi A Pellegrino N Di Stefano V Raucci U Luchetti A Parisi. Misdiagnosis and pitfalls in Panayiotopoulos syndrome. Epilepsy Behav. (2019) 98:124–8. 10.1016/j.yebeh.2019.07.016 31369969

[72] Gelfand AA Episodic syndromes that may be associated with migraine: A.K.A. the childhood periodic syndromes. Headache. (2015) 55:1358–64. 10.1111/head.12624 26234380 PMC4715532

[73] Dignan F Symon DN AbuArafeh I Russell G. The prognosis of cyclical vomiting syndrome. Arch Dis Child. (2001) 84:55–7. 10.1136/adc.84.1.55 11124785 PMC1718619

[74] Jones MP Dilley JB Drossman D Crowell MD. Brain-gut connections in functional GI disorders: anatomic and physiologic relationships. Neurogastroenterol Motil. (2006) 18:91–103. 10.1111/j.1365-2982.2005.00730.x 16420287

[75] van Driessche A Sermijn E Paemeleire K van Coster R Vogelaers D. Cyclic vomiting syndrome: case report and short review of the literature. Acta Clin Belg. (2012) 67:123–6. 10.2143/ACB.67.2.2062642 22712168

[76] Tarantino S Capuano A Torriero R Citti M Vollono C Gentile S. Migraine equivalents as part of migraine syndrome in childhood. Pediatr Neurol. (2014) 51:645–9. 10.1016/j.pediatrneurol.2014.07.018 25155656

[77] Gelfand AA. Migraine and childhood periodic syndromes in children and adolescents. Curr Opin Neurol. (2013) 26:262–8. 10.1111/jcap.12040 23549418

[78] Tarantino S de Ranieri C Dionisi C Gagliardi V Capuano A Vigevano F. Migraine equivalents and related symptoms, psychological profile and headache features: which relationship? J Headache Pain. (2015) 16:536. 10.1186/s10194-015-0536-2 26059348 PMC4467804

[79] Li BUK. Managing cyclic vomiting syndrome in children: beyond the guidelines. Eur J Pediatr. (2018) 177:1435–42. 10.1007/s00431-018-3218-7 30076469 PMC6153591

[80] Tarbell SE Li BU. Anxiety measures predict health-related quality of life in children and adolescents with cyclic vomiting syndrome. J Pediatr. (2015) 167:633–8.e1. 10.1016/j.jpeds.2015.05.032 26095286

[81] Wang-Hall J Li BUK Tarbell SE. Family health-related quality of life in pediatric cyclic vomiting syndrome. J Pediatr Gastroenterol Nutr. (2018) 66:738–43. 10.1097/MPG.0000000000001797 29697487

[82] Chelimsky TC Chelimsky GG. Autonomic abnormalities in cyclic vomiting syndrome. J Pediatr Gastroenterol Nutr. (2007) 44:326–30. 10.1097/MPG.0b013e31802bddb7 17325552

[83] Pareek N Fleisher DR Abell T. Cyclic vomiting syndrome: what a gastroenterologist needs to know. Am J Gastroenterol. (2007) 102:2832–40. 10.1111/j.1572-0241.2007.01549.x 18042112

[84] Cutrer FM Charles A. The neurogenic basis of migraine. Headache. (2008) 48:1411–4. 10.1111/j.1526-4610.2008.01277.x 19006559

[85] Boles RG Powers AL Adams K. Cyclic vomiting syndrome plus. J Child Neurol. (2006) 21:182–8. 10.2310/7010.2006.00040 16901417

[86] Kaul A Kaul KK. Cyclic vomiting syndrome: a functional disorder. Pediatr Gastroenterol Hepatol Nutr. (2015) 18:224–9. 10.5223/pghn.2015.18.4.224 26770896 PMC4712534

[87] Panayiotopoulos CP. Vomiting as an ictal manifestation of epileptic seizures and syndromes. J Neurol Neurosurg Psychiatry. (1988) 51:1448–51. 3148690 10.1136/jnnp.51.11.1448 PMC1032819

[88] Shuper A Goldberg-Stern H. Ictus emeticus (ictal vomiting). Pediatr Neurol. (2004) 31:283–6. 10.1016/j.pediatrneurol.2004.04.013 15464642

[89] Covanis A Panayiotopoulos syndrome: a benign childhood autonomic epilepsy frequently imitating encephalitis, syncope, migraine, sleep disorder, or gastroenteritis. Pediatrics. (2006) 118:e1237–43. 10.1542/peds.2006-0623 16950946

[90] Carbonari G Tonti G Di Pisa V Franzoni E Cordelli DM. Pediatric epilepsies misdiagnosed as gastrointestinal disorders. Epilepsy Behav. (2018) 83:137–9. 10.1016/j.yebeh.2018.03.034 29705623

[91] McAbee GN Morse AM Cook W Tang V Brosgol Y. Neurological etiologies and pathophysiology of cyclic vomiting syndrome. Pediatr Neurol. (2020) 106:4–9. 10.1016/j.pediatrneurol.2019.12.001 32107138

[92] Sorensen CJ DeSanto K Borgelt L Phillips KT Monte AA Cannabinoid hyperemesis syndrome: diagnosis, pathophysiology,and treatment-a systematic review. J Med Toxicol. (2017) 13:71–87. 10.1007/s13181-016-0595-z 28000146 PMC5330965

[93] Enokizono T Nemoto K Fujiwara J Tanaka R Ohto T. Cyclic vomiting syndrome after acute autonomic and sensory neuropathy. Pediatr Int. (2017) 59:503–5. 10.1111/ped.13232 28244649

[94] Aylward SC Reem RE. Pediatric intracranial hypertension. Pediatr Neurol. (2017) 66:32–43. 10.1016/j.pediatrneurol.2016.08.010 27940011

[95] Baumgartner MR Hörster F Dionisi-Vici C Haliloglu G Karall D Chapman KA. Proposed guidelines for the diagnosis and management of methylmalonic and propionic acidemia. Orphanet J Rare Dis. (2014) 9:130. 10.1186/s13023-014-0130-8 25205257 PMC4180313

[96] Häberle J Burlina A Chakrapani A Dixon M Karall D Linder M. Suggested guidelines for the diagnosis and management of urea cycle disorders: First revision. J Inherit Metab Dis. (2019) 42:1192–230. 10.1002/jimd.12100 30982989

[97] Kölker S Christensen E Leonard JV Greenberg CR Boneh A Burlina AB. Diagnosis and management of glutaric aciduria type I–revised recommendations. J Inherit Metab Dis. (2011) 34:677–94. 10.1007/s10545-011-9289-5 21431622 PMC3109243

[98] Heringer J Boy SP Ensenauer R Assmann B Zschocke J Harting I. Use of guidelines improves the neurological outcome in glutaric aciduria Type I. Ann Neurol. (2010) 68:743–52. 10.1002/ana.22095 21031586

[99] Anderson KE Bloomer JR Bonkovsky HL Kushner JP Pierach CA Pimstone NR. Recommendations for the diagnosis and treatment of the acute porphyrias. Ann Intern Med. (2005) 142:439–50. 10.7326/0003-4819-142-6-200503150-00010 15767622

[100] Sass JO. Inborn errors of ketogenesis and ketone body utilization. J Inherit Metab Dis. (2012) 35:23–8. 10.1007/s10545-011-9324-6 21479626

[101] van Hasselt PM Ferdinandusse S Monroe GR Ruiter JPN Turkenburg M Geerlings MJ. Monocarboxylate transporter 1 deficiency and ketone utilization. N Engl J Med. (2014) 371:1900–7. 10.1056/NEJMoa1407778 25390740

[102] Demirbas D Brucker WJ Berry GT. Inborn errors of metabolism with hepatopathy: metabolism defects of galactose, fructose, and tyrosine. Pediatr Clin North Am. (2018) 65:337–52. 10.1016/j.pcl.2017.11.008 29502917

[103] Barkaoui E Debray D Habès D Ogier H Bernard O. [Favorable outcome of treatment with NTBC of acute liver insufficiency disclosing hereditary tyrosinemia type I]. Arch Pediatr. (1999) 6:540–4. 10.1016/S0929-693X(99)80562-4 10370811

[104] Li BU Fleisher DR. Cyclic vomiting syndrome: features to be explained by a pathophysiologic model. Dig Dis Sci. (1999) 44:S13–8. 10490033

[105] Pfau BT Li BU Murray RD Heitlinger LA McClung HJ Hayes JR. Differentiating cyclic from chronic vomiting patterns in children: quantitative criteria and diagnostic implications. Pediatrics. (1996) 97:364–8. 8604272

[106] Haan J Kors EE Ferrari MD. Familial cyclic vomiting syndrome. Cephalalgia. (2002) 22:552. 10.1046/j.1468-2982.2002.00420.x 12230597

[107] Salpietro CD Briuglia S Merlino MV. A mitochondrial DNA mutation (A3243G mtDNA) in a family with cyclic vomiting. Eur J Pediatr. (2003) 162:727–8. 10.1007/s00431-003-1280-1 12905015

[108] Boles RG Adams K Ito M Li BU. Maternal inheritance in cyclic vomiting syndrome with neuromuscular disease. Am J Med Genet A. (2003) 120A:474–82. 10.1002/ajmg.a.20126 12884425

[109] Boles RG Adams K Li BU. Maternal inheritance in cyclic vomiting syndrome. Am J Med Genet. (2005) 133A:71–7. 10.1002/ajmg.a.30524 15643622

[110] Rinaldo P. Mitochondrial fatty acid oxidation disorders and cyclic vomiting syndrome. Dig Dis Sci. (1999) 44(8 Suppl.):S97–102. 10490047

[111] Boles RG Baldwin EE Prezant TR. Combined cyclic vomiting and Kearns-Sayre syndromes. Pediatr Neurol. (2007) 36:135–6. 10.1016/j.pediatrneurol.2006.09.008 17275670

[112] Venkatesan T Zaki EA Kumar N Sengupta J Ali M Malik B. Quantitative pedigree analysis and mitochondrial DNA sequence variants in adults with cyclic vomiting syndrome. BMC Gastroenterol. (2014) 14:181. 10.1186/1471-230X-14-181 25332060 PMC4287476

[113] Boles RG Zaki EA Kerr JR Das K Biswas S Gardner A. Increased prevalence of two mitochondrial DNA polymorphisms in functional disease: Are we describing different parts of an energy-depleted elephant? Mitochondrion. (2015) 23:1–6. 10.1016/j.mito.2015.04.005 25934187

[114] Nozaki F Kusunoki T Okamoto N Yamamoto Y Miya F Tsunoda T. ALDH18A1-related cutis laxa syndrome with cyclic vomiting. Brain Dev. (2016) 38:678–84. 10.1016/j.braindev.2016.01.003 26829900

[115] OMIM ^TM^ Online Mendelian Inheritance in Man. An Online Catalog of Human Genes and Genetic Disorders. (2020). Available online at: https://omim.org. (accessed June 26, 2020).

[116] Boles RG Williams JC. Mitochondrial disease and cyclic vomiting syndrome. Dig Dis Sci. (1999) 44 (8 Suppl.):S103–7. 10.1016/S0039-6257(99)00087-9 10490048

[117] Boles RG Chun N Senadheera D Wong LJ. Cyclic vomiting syndrome and mitochondrial DNA mutations. Lancet. (1997) 350:1299–300. 10.1016/S0140-6736(05)62477-4 9357417

[118] Wang Q Ito M Adams K Li BU Klopstock T Maslim A. Mitochondrial DNA control region sequence variation in migraine headache and cyclic vomiting syndrome. Am J Med Genet A. (2004) 131:50–8. 10.1002/ajmg.a.30323 15368478

[119] Boles RG Zaki EA Lavenbarg T Hejazi R Foran P Freeborn J. Are pediatric and adult-onset cyclic vomiting syndrome (CVS) biologically different conditions? Relationship of adult-nset CVS with the migraine pediatric CVS associated common mtDNA polymorphisms 16519T 3010A. Neurogastroenterol Motil. (2009) 21:936–e72. 10.1111/j.1365-2982.2009.01305.x 19368653

[120] Finsterer J Hayman J. Mitochondrial disorder caused Charles Darwin's cyclic vomiting syndrome. Int J Gen Med. (2014) 7:59–70. 10.2147/IJGM.S54846 24453499 PMC3892961

[121] Ye Z Xue A Huang Y Wu Q. Children with cyclic vomiting syndrome: phenotypes, disease burden and mitochondrial DNA analysis. BMC Gastroenterol. (2018) 18:104. 10.1186/s12876-018-0836-5 29969994 PMC6029397

[122] Wasilewski A Lewandowska U Mosinska P Watala C Storr M„ Fichna J. Cannabinoid receptor type 1 and mu-opioid receptor polymorphisms are associated with cyclic vomiting syndrome. Am J Gastroenterol. (2017) 112:933–9. 10.1038/ajg.2017.73 28349993

[123] Marx SO Reiken S Hisamatsu Y Jayaraman T Burkhoff D Rosemblit N. PKA phosphorylation dissociates FKBP12.6 from the calcium release channel (ryanodine receptor): defective regulation in failing hearts. Cell. (2000) 101:365–76. 10.1016/S0092-8674(00)80847-8 10830164

[124] Spiller TR Künzler K Caduff B. Cyclic vomiting syndrome: an important differential diagnosis of cannabinoid hyperemesis syndrome. BMJ. (2019). 366:l5615. 10.1136/bmj.l5615 31548258

[125] Venkatesan T Hillard CJ Rein L Banerjee A Lisdahl K. Patterns of cannabis use in patients with cyclic vomiting syndrome. Clin Gastroenterol Hepatol. (2020) 18:1082–90.e2. 10.1016/j.cgh.2019.07.039 31352091

[126] Venkatesan T Levinthal DJ Li BUK Tarbell SE Adams KA Issenman RM. Role of chronic cannabis use: cyclic vomiting syndrome vs cannabinoid hyperemesis syndrome. Neurogastroenterol Motil. (2019) 31 (Suppl. 2):e13606. 10.1111/nmo.13606 31241817 PMC6788295

[127] Kakisaka Y Wakusawa K Sato I Haginoya K Uematsu M Hirose M. Successful treatment with sumatriptan in a case with cyclic vomiting syndrome combined with 18q-syndrome. J Child Neurol. (2009) 24:1561–3. 10.1177/0883073809334384 19794103

[128] Chew S Balasubramanian R Chan WM Kang PB Andrews C Webb BD. A novel syndrome caused by the E410K amino acid substitution in the neuronal β-tubulin isotype 3. Brain. (2013) 136(Pt 2):522–35. 10.1093/brain/aws345 23378218 PMC3572929

[129] Normandin PA. Pediatric emergency update: cyclic vomiting syndrome. J Emerg Nurs. (2015) 41:260–2; quiz 269. 10.1016/j.jen.2015.03.003 25814094

[130] Venkatesan T Tarbell S Adams K McKanry J Barribeau T Beckmann K. A survey of emergency department use in patients with cyclic vomiting syndrome. BMC Emerg Med. (2010) 10:4. 10.1186/1471-227X-10-4 20181253 PMC2841069

[131] Di Lorenzo C Approach to the Infant or Child with Nausea and Vomiting. Up to Date. (2020). aVhttps://www.uptodate.com/contents/approach-to-the-infant-or-child-ailable online at: with-nausea-and-vomiting (accessed May 12, 2020).

[132] Venkatesan T Levinthal DJ Tarbell SE Jaradeh SS Hasler WL Issenman RM. Guidelines on management of cyclic vomiting syndrome in adults by the American neurogastroenterology and motility society and the cyclic vomiting syndrome association. Neurogastroenterol Motil. (2019) 31 (Suppl 2):e13604. 10.1111/nmo.13604 31241819 PMC6899751

[133] Raucci U Pro S Di Capua M Di Nardo G Villa MP Striano P. A reappraisal of the value of video-EEG recording in the emergency department. Expert Rev Neurother. (2020) 20:459–75. 10.1080/14737175.2020.1747435 32249626

[134] Gui S Patel N Issenman R Kam AJ Acute management of pediatric cyclic vomiting syndrome: a systematic review. J Pediatr. (2019) 214:158–64. e4. 10.1016/j.jpeds.2019.06.057 31540764

[135] Hikita T Kodama H Kaneko S Amakata K Ogita K Mochizuki D. Sumatriptan as a treatment for cyclic vomiting syndrome: a clinical trial. Cephalalgia. (2011) 31:504–7. 10.1177/0333102410390398 21147834

[136] Cristofori F Thapar N Saliakellis E Kumaraguru N Elawad M Kiparissi F. Efficacy of the neurokinin-1 receptor antagonist aprepitant in children with cyclical vomiting syndrome. Aliment Pharmacol Ther. (2014) 40:309–17. 10.1111/apt.12822 24898244

[137] Slutsker B Konichezky A Gothelf D. Breaking the cycle: cognitive behavioral therapy and biofeedback training in a case of cyclic vomiting syndrome. Psychol Health Med. (2010) 15:625–31. 10.1080/13548506.2010.498893 21154016

[138] Khasawinah TA Ramirez A Berkenbosch JW Tobias JD. Preliminary experience with dexmedetomidine in the treatment of cyclic vomiting syndrome. Am J Ther. (2003) 10:303–7. 10.1097/00045391-200307000-00012 12845396

[139] Millichap JG Yee MM. The diet factor in pediatric and adolescent migraine. Pediatr Neurol. (2003) 28:9–15. 10.1016/s0887-8994(02)00466-6 12657413

[140] Lucarelli S Corrado G Pelliccia A D'Ambrini G Cavaliere M Barbato M. Cyclic vomiting syndrome and food allergy/intolerance in seven children: a possible association. Eur J Pediatr. (2000) 159:360–3. 10.1007/s004310051287 10834522

[141] Lewis DW Yonker M Winner P Sowell M. The treatment of pediatric migraine. Pediatr Ann. (2005) 34:448–60. 10.1016/S0147-9563(05)00187-1 16018227

[142] Allen JH de Moore GM Heddle R Twartz JC. Cannabinoid hyperemesis: cyclical hyperemesis in association with chronic cannabis abuse. Gut. (2004) 53:1566–70. 10.1136/gut.2003.036350 15479672 PMC1774264

[143] Saxena PR DenBoer MO. Pharmacology of antimigraine drugs. J Neurol. (1991) 238(Suppl. 1):S28–35. 10.1007/BF01642903 1646288

[144] Krasaelap A Madani S. Cyproheptadine: a potentially effective treatment for functional gastrointestinal disorders in children. Pediatr Ann. (2017) 46:e120–5. 10.3928/19382359-20170213-01 28287686

[145] Andersen JM Sugerman KS Lockhart JR Weinberg WA Effective prophylactic therapy for cyclic vomiting syndrome in children using amitriptyline or cyproheptadine. Pediatrics. (1997) 100:977–81. 10.1542/peds.100.6.977 9374568

[146] Madani S Cortes O Thomas R. Cyproheptadine use in children with functional gastrointestinal disorders. J Pediatr Gastroenterol Nutr. (2016) 62:409–13. 10.1097/MPG.0000000000000964 26308312

[147] Badihian N Saneian H Badihian S Yaghini O. Prophylactic therapy of cyclic vomiting syndrome in children: comparison of amitriptyline and cyproheptadine: a randomized clinical trial. Am J Gastroenterol. (2018) 113:135–40. 10.1038/ajg.2017.194 28719594

[148] Salmon MA Walters DD. Pizotifen in the prophylaxis of cyclical vomiting. Lancet. (1985) 1:1036–7. 10.1016/S0140-6736(85)91630-7 2859478

[149] Haghighat M Dehghani SM Shahramian I Imanieh MH Teimouri A Noori NM. Combination of erythromycin and propranolol for treatment of childhood cyclic vomiting syndrome: a novel regimen. Gastroenterol Hepatol Bed Bench. (2015) 8:270–7. 26468347 PMC4600517

[150] Haghighat M Memari H Honar N Dehghani SM Imanieh MH Injoo SJ. The efficacy and duration of treatment with propranolol in children with cyclic vomiting syndrome in southern Iran. Prz Gastroenterol. (2017) 12:291–5. 10.5114/pg.2017.72105 29358999 PMC5771454

[151] Vanderhoof JA Young R Kaufman SS Ernst L. Treatment of cyclic vomiting in childhood with erythromycin. J Pediatr Gastroenterol Nutr. (1995) 21(Suppl. 1):S60–2. 8708873 10.1097/00005176-199501001-00017

[152] Gore L Chawla S Petrilli A Hemenway M Schissel D Chua V. Aprepitant in adolescent patients for prevention of chemotherapy-induced nausea and vomiting: a randomized, double-blind, placebo-controlled study of efficacy and tolerability. Pediatr Blood Cancer. (2009) 52:242–7. 10.1002/pbc.21811 18985740

[153] Albany C Brames MJ Fausel C Johnson CS Picus J Einhorn LH. Randomized, double-blind, placebo-controlled, phase III cross-over study evaluating the oral neurokinin-1 antagonist aprepitant in combination with a 5HT3 receptor antagonist and dexamethasone in patients with germ cell tumors receiving 5-day cisplatin combination chemotherapy regimens: a hoosier oncology group study. J Clin Oncol. (2012) 30:3998–4003. 10.1200/JCO.2011.39.5558 22915652

[154] Treepongkaruna S Jarasvaraparn C Tanpowpong P Lertudomphonwanit C. Short-and long term outcomes of children with cyclic vomiting syndrome. J Med Assoc Thai. (2014) 97:1077–83. 25632624

[155] Bagherian Z Yaghini O Saneian H Badihian S. Comparison of the efficacy of amitriptyline and topiramate in prophylaxis of cyclic vomiting syndrome. Iran J Child Neurol. (2019) 13:37–44. 30613204 PMC6296701

[156] Herlihy JD Reddy S Shanker A McCallum R. Cyclic vomiting syndrome: an overview for clinicians. Expert Rev Gastroenterol Hepatol. (2019) 13:1137–43. 10.1080/17474124.2019.1691527 31702939

[157] Gokhale R Huttenlocher PR Brady L Kirschner BS. Use of barbiturates in the treatment of cyclic vomiting during childhood. J Pediatr Gastroenterol Nutr. (1997) 25:64–7. 9226529 10.1097/00005176-199707000-00010

[158] Olmez A Köse G Turanli G. Cyclic vomiting with generalized epileptiform discharges responsive to topiramate therapy. Pediatr Neurol. (2006) 35:348–51. 10.1016/j.pediatrneurol.2006.06.014 17074606

[159] Hikita T Kodama H Nakamoto N Kaga F Amakata K Ogita K. Effective prophylactic therapy for cyclic vomiting syndrome in children using valproate. Brain Dev. (2009) 31:411–3. 10.1016/j.braindev.2008.07.005 18752910

[160] Sezer OB Sezer T. A New Approach to the Prophylaxis of Cyclic Vomiting: Topiramate. J Neurogastroenterol Motil. (2016) 22:656–60. 10.5056/jnm16035 27302967 PMC5056575

[161] Victor S Ryan SW. Drugs for preventing migraine headaches in children. Cochrane Database Syst Rev. (2014) 2014:CD002761. 10.1002/14651858.CD002761.pub2 25019292 PMC6464504

[162] Spierings EL. Mechanism of migraine and action of antimigraine medications. Med Clin North Am. (2001) 85:943–58, vi–vii. 10.1016/s0025-7125(05)70352-7 11480266

[163] Kothare SV. Efficacy of flunarizine in the prophylaxis of cyclical vomiting syndrome and abdominal migraine. Eur J Paediatr Neurol. (2005) 9:23–6. 10.1016/j.ejpn.2004.11.002 15701563

[164] Martinez-Esteve Melnikova A Schäppi MG Korff C. Riboflavin in cyclic vomiting syndrome: efficacy in three children. Eur J Pediatr. (2016) 175:131–5. 10.1007/s00431-015-2597-2 26226892

[165] Hassani MEME Saad B Mounir M Kouach J Rahali DM. Catamenial cyclic vomiting syndrome responding to oestrogen therapy: an adolescent case report. Pan Afr Med J. (2019) 33:286. 10.11604/pamj.2019.33.286.17978 31692884 PMC6815497

[166] Rusy LM Weisman SJ Hainsworth KR. Developing an in-patient a acupuncture treatment in a pediatric hospital. J Complement Integr Med. (2013). 10.1515/jcim-2012-0056 23652642

[167] Fennig S Fennig S Cyclic vomiting syndrome: role of a psychiatric inpatient unit in a general children's hospital. J Pediatr Gastroenterol Nutr. (1999) 29:207–10. 10435661 10.1097/00005176-199908000-00020

[168] Sikand M Sharma P. Psychological intervention in cyclic vomiting syndrome in adolescents: a case series. J Child Adolesc Ment Health. (2019) 3:182–8. 10.2989/17280583.2019.1674660 31805841

